# The influence of stress on the neural underpinnings of disinhibited eating: a systematic review and future directions for research

**DOI:** 10.1007/s11154-023-09814-4

**Published:** 2023-06-13

**Authors:** Emily Giddens, Brittany Noy, Trevor Steward, Antonio Verdejo-García

**Affiliations:** 1 Turner Institute for Brain and Mental Health, Faculty of Medicine, Nursing and Health Sciences, Monash University, 18 Innovation Walk, Clayton, VIC 3800 Australia; 2 Melbourne School of Psychological Sciences, Faculty of Medicine, Dentistry and Health Sciences, University of Melbourne, Parkville, VIC Australia; 3 Melbourne Neuropsychiatry Centre, Department of Psychiatry, The University of Melbourne, Melbourne, VIC Australia

**Keywords:** Disinhibited eating, Binge eating, Acute stress, Chronic stress, Obesity, fMRI

## Abstract

Disinhibited eating involves overconsumption and loss of control over food intake, and underpins many health conditions, including obesity and binge-eating related disorders. Stress has been implicated in the development and maintenance of disinhibited eating behaviours, but the mechanisms underlying this relationship are unclear. In this systematic review, we examined how the impact of stress on the neurobiological substrates of food-related reward sensitivity, interoception and cognitive control explains its role in disinhibited eating behaviours. We synthesised the findings of functional magnetic resonance imaging studies including acute and/or chronic stress exposures in participants with disinhibited eating. A systematic search of existing literature conducted in alignment with the PRISMA guidelines identified seven studies investigating neural impacts of stress in people with disinhibited eating. Five studies used food-cue reactivity tasks, one study used a social evaluation task, and one used an instrumental learning task to probe reward, interoception and control circuitry. Acute stress was associated with deactivation of regions in the prefrontal cortex implicated in cognitive control and the hippocampus. However, there were mixed findings regarding differences in reward-related circuitry. In the study using a social task, acute stress associated with deactivation of prefrontal cognitive control regions in response to negative social evaluation. In contrast, chronic stress was associated with both deactivation of reward and prefrontal regions when viewing palatable food-cues. Given the small number of identified publications and notable heterogeneity in study designs, we propose several recommendations to strengthen future research in this emerging field.

**Supplementary Information:**

The online version contains supplementary material available at 10.1007/s11154-023-09814-4.

## Introduction

Disinhibited eating (DE) refers to a range of maladaptive eating behaviours characterised by overconsumption (i.e., eating beyond the point of satiety or metabolic drive) and loss of control in the presence of palatable or energy-dense foods [1, 2]. DE broadly captures behaviours such as stress/emotional eating, binge-eating, and grazing/snacking in the absence of hunger [1]. Such behaviours have been implicated in both the development and maintenance of higher body weight (i.e., body mass index [BMI] > 25 kg/m^2^) [3, 4], as well as binge eating-spectrum eating disorders, such binge eating disorder (BED), bulimia nervosa (BN) and anorexia nervosa binge-purge subtype (AN-BP) [1, 5, 6]. However, both BN and AN-BP are less frequently associated with excess weight than BED given they are also characterised by compensatory behaviours (i.e., purging). Relative to the general population, individuals who experience DE are also at greater risk of developing mood, anxiety, and substance-use disorders [4, 7–9], as well as greater interpersonal distress [11] and overall poorer quality of life [12, 13]. Despite higher body weight and BED being characterised by DE, both conditions are often managed in isolation. In particular, treatments for managing higher weight remain predominantly focused on promoting weight-loss, rather than addressing potential underlying neurocognitive mechanisms [10, 11]. Likewise, common therapeutic approaches for binge-eating spectrum disorders (i.e., cognitive behavioural therapy) do not efficiently facilitate weight-loss [12]. Provided both excess weight and binge eating-spectrum disorders in themselves are highly comorbid [5], better understanding and management of the mechanisms underlying DE may provide a more efficient, transdiagnostic method of treatment for both higher weight and BED.

One mechanism that has been strongly implicated in the development and maintenance of DE is stress. Stress refers to the triggering of several affective, cognitive and physiological processes by perceived threats (stressors) [13, 14]. While these processes aim to safely overcome perceived stressors, prolonged or inappropriate triggering of the stress response is often detrimental to psychological and physical wellbeing [15–18]. Individuals who experience heightened levels of DE (i.e., BED, emotional eating, etc.) appear more sensitive to the effects of stress, including greater levels of perceived life stress [8, 19–21], poorer ability to cope with stressors [22–24], and greater negative affect (i.e., depressed mood) when stressed [9, 25]. Furthermore, relative to healthy controls, people who experience DE are more likely to engage in unrestrained food-consumption following exposure to both acute laboratory and real-life stressors [26–33]. DE episodes in themselves can also be significantly distressing [34], and in turn may perpetuate future episodes of DE if not adequately managed. Overall, stress, both proximate and chronic, appear to play an integral role in the development and precipitation of DE and its associated behaviours, and as such may present as a useful treatment target for managing higher weight and binge-eating spectrum disorders. However, the exact mechanism through which stress exacerbates DE currently remains unclear.

Theoretical models of stress and DE suggest that this relationship may occur for several reasons. Affective-regulation models posit that individuals prone to DE lack adaptive methods of overcoming negative mood, and in turn consume foods that are rewarding (i.e., palatable, energy-dense) to alleviate negative mood-states induced by stressful events [35–37]. Stress-induced DE is therefore reinforced by the subsequent alleviation of negative mood. However, the alleviation of negative mood states is generally transient, and in turn individuals may become reliant in DE to regulate mood in response to stressful situations. Similarly, acquired-preparedness models suggest those who experience DE are predisposed to impulsive traits (i.e., negative urgency), and in turn maladaptively associate impulsive actions (i.e., eating large quantities of food) with the alleviation of negative mood states [38, 39]. Physiological accounts, rather, suggest that prolonged activation of the physiological stress response (i.e., hypothalamic-pituitary-adrenal [HPA] axis reactivity) alters metabolism and food preference, biasing food-choice towards foods high in energy content as an evolutionary means of enhancing available energy stores to overcome potential stressors [40, 41]. Dysregulation of the HPA axis response may in turn heighten preference for energy-dense foods, even in the absence of immediate stressors.

Given the paucity of evidence indicating stress exacerbates DE, there has been growing impetus to better understand whether these theoretical accounts are empirically supported by clear biological mechanisms. To date, most research attempting to identify such mechanisms has largely focused on the role of HPA-axis, namely investigating cortisol reactivity. Indeed, several studies [26, 27] have demonstrated that young people and adults who experience binge-eating demonstrate heightened basal cortisol relative to controls, which is consistent with chronic HPA-axis activation [42]. Furthermore, behavioural studies by Rouach et al. [43] and Van Strien et al. [44] identified acute laboratory stress is associated with blunted post-stress cortisol reactivity and greater *ad libitum* food consumption in individuals with BED and emotional eating, respectively, potentially indicating downregulation of the HPA-axis due to chronic activation [45]. However, these results have not been consistently observed, with other studies identifying a non-significant, but trending increases in cortisol following acute stress induction in those with DE relative to controls [26, 46]. Furthermore, several studies have failed to identify any significant differences between basal and acute post-stress cortisol levels between obese women with BED and weight-matched controls, however significant changes in post-stress *ad libitum* food consumption were observed in those with BED [47, 48]. Taken together, current findings regarding cortisol reactivity and its role in DE remain inconsistent and have not yet effectively parsed the effects of weight-status and DE on cortisol reactivity.

Considering the limitations of existing research, there has been growing interest in other neurobiological mechanisms that may explain the link between stress and DE. One emerging area of research is interested in elucidating how functional brain activity may contribute to the exacerbation of DE under stressful conditions. Existing functional neuroimaging literature suggests that populations characterised by DE demonstrate hyperactivity in mesocorticolimbic regions (i.e., striatum, ventral tegmental area, amygdala) in response to energy-dense, palatable food-cues [49–52], accompanied by diminished recruitment of prefrontal regions implicated in inhibitory control (i.e., inferior frontal gyrus) [53], and regulation of other cognitive processes underpinning emotional / social processing, and decision-making (i.e., ventromedial prefrontal cortex, dorsolateral prefrontal cortex) [50, 54]. Furthermore, it has been suggested that individuals with DE display decreased recruitment of key regions of the salience (mid cingulo-insular) network, such as the anterior insula [53–56], which integrates internal signals of emotional and physiological states (i.e., interoception), and the anterior cingulate cortex (ACC) [57, 58], which is involved in emotional regulation, attentional allocation, and error-monitoring. Networks implicated in DE are also involved in stress reactivity. For example, acute stress is associated with attenuation in prefrontal regions implicated in cognitive control (i.e., dlPFC) and increased recruitment of the salience network during cognitively demanding tasks [59, 60]. Moreover, acute stress is associated with increased recruitment of the mesocorticolimbic regions, such as the ventral striatum during reward processing [61]. Chronic stress is also associated with dysregulation of mesocorticolimbic circuitry [62], and decreased recruitment of dorsolateral prefrontal cortex, insula and anterior cingulate cortex [60]. Provided the overlap between networks implicated in DE and stress reactivity, it has been hypothesised that exacerbation of DE symptoms in response to stress may reflect a synergistic effect of the neural stress response coupled with vulnerabilities in brain regions implicated in reward processing, cognitive control, and interoception [52].

Therefore, the current review aims to systematically investigate the emerging functional magnetic resonance imaging (fMRI) studies examining the effects of stress on brain activity in populations characterised by DE, provide insight into the current methodological practices in this field, and finally to address any potential limitations of existing work and provide suggestions for addressing these concerns. Based on behavioural research of stress and DE, as well as fMRI research of DE populations under neutral conditions, we hypothesise that both acute and chronic stress will be associated with (1) increased activity in the mesocorticolimbic reward network, (2) decreased recruitment of prefrontal regions implicated in cognitive control, and (3) increased activity in regions of the salience network, in individuals with DE relative to healthy controls.

## Methods

The systematic review was conducted in accordance with recommendations outlined in the PRISMA statement [54]. The objectives and protocol used were pre-registered in Open Science Framework (https://osf.io/97sm3/).

### Search strategy

Database searchers were conducted up to 03/01/2022. No limits were set for the publication dates of articles. Searches were conducted in the following electronic databases: MEDLINE Ovid® PsychInfo Ovid®, Scopus, CINAHL, and Web of Science Core Collection. Reference lists of eligible articles were manually searched for to identify any further relevant studies. A summary of search terms is outlined in Table 1, with the specific search strategies used and result output from each database available in Supplementary Table 1. Keywords were selected via Medical Subject Headings (MEsH) for each relevant construct (i.e., acute stress, disinhibited eating, neuroimaging). Furthermore, related terms for disinhibited eating outlined by Vainik et al. [4] were also used to inform keywords for the ‘disinhibited eating’ construct.

**Table 1:** Key search terms by construct used in database searches

Construct	Keywords
Disinhibited eating	“binge eating” OR “binge eating disorder” OR “binge eating scale” OR “eating disorder examination questionnaire” OR “subthreshold bing*” OR “emotional eating” OR “uncontrol* eating” OR “loss of control” OR “LOC” OR “opportunistic eating” OR “external eating” OR “disinhibition” OR “disinhibited eating” OR food craving” OR “hedonic eating” OR hedonic hunger” OR “reward-based eating”.
Stress	“acute stress*” OR “daily stress*” OR “transient stress*” OR “cortisol” OR “stress test” OR “stress paradigm” OR “negative affect*” OR “Trier Social Stress Test” OR “Cold Pressor Test” OR “arithmetic task” OR “interpersonal stress” OR “social evaluation” OR “HPA” OR “hypothalamic pituitary adrenal” OR “chronic stress*” OR “hair cortisol” or “saliv* cortisol” OR “cumulative stress*” OR “allostasis” OR “allostatic load”.
Functional neuroimaging	functional neuroimag*” or “fMRI” or “MRI” or “white matter” OR “gr?y matter” or “brain volume” or “BOLD”.

### Eligibility criteria

Eligibility was considered in accordance with the PICOS criteria outlined in Liberati et al. [54]. Eligible studies were required to fit the following criteria: (1) original, peer-reviewed articles published or in press in a peer-reviewed journal; (2) English-language articles only; (3) participants with disinhibited eating behaviours (identified either by a formal diagnosis of BED, or threshold scores on a validated measure of disinhibited eating or binge eating, such as the Binge Eating Scale [55]; 4) inclusion of a validated acute stress induction paradigm, such as the Trier Social Stress Test [56], or an element of these paradigms (i.e., social evaluation), or a validated measure of chronic stress (i.e., hair cortisol); and 5) studies must include a fMRI as an outcome.

Articles were not considered for review if they included non-human individuals or human participants younger than 12 years of age, given the onset of disinhibited eating tends to peak in adolescence [57]. Furthermore, studies utilising electrophysiology methods (i.e., electroencephalography) were excluded. Participants who exhibit disinhibited eating in the presence of maladaptive compensatory behaviours (i.e., bulimia nervosa) were also excluded given our focus on disinhibited eating patterns alone. Moreover, as disinhibited eating is associated with increased presence of mood and anxiety disturbances, the inclusion of selective comorbid mood or anxiety disorders / symptoms were permitted for individuals with disinhibited eating only. Other psychiatric comorbidities (i.e., substance-use disorders, personality disorders) were grounds for exclusion across both individuals with disinhibited eating and healthy controls. Finally, participants with any neurological or metabolic illnesses were also excluded.

### Study selection and data collection

**Fig. 1:**
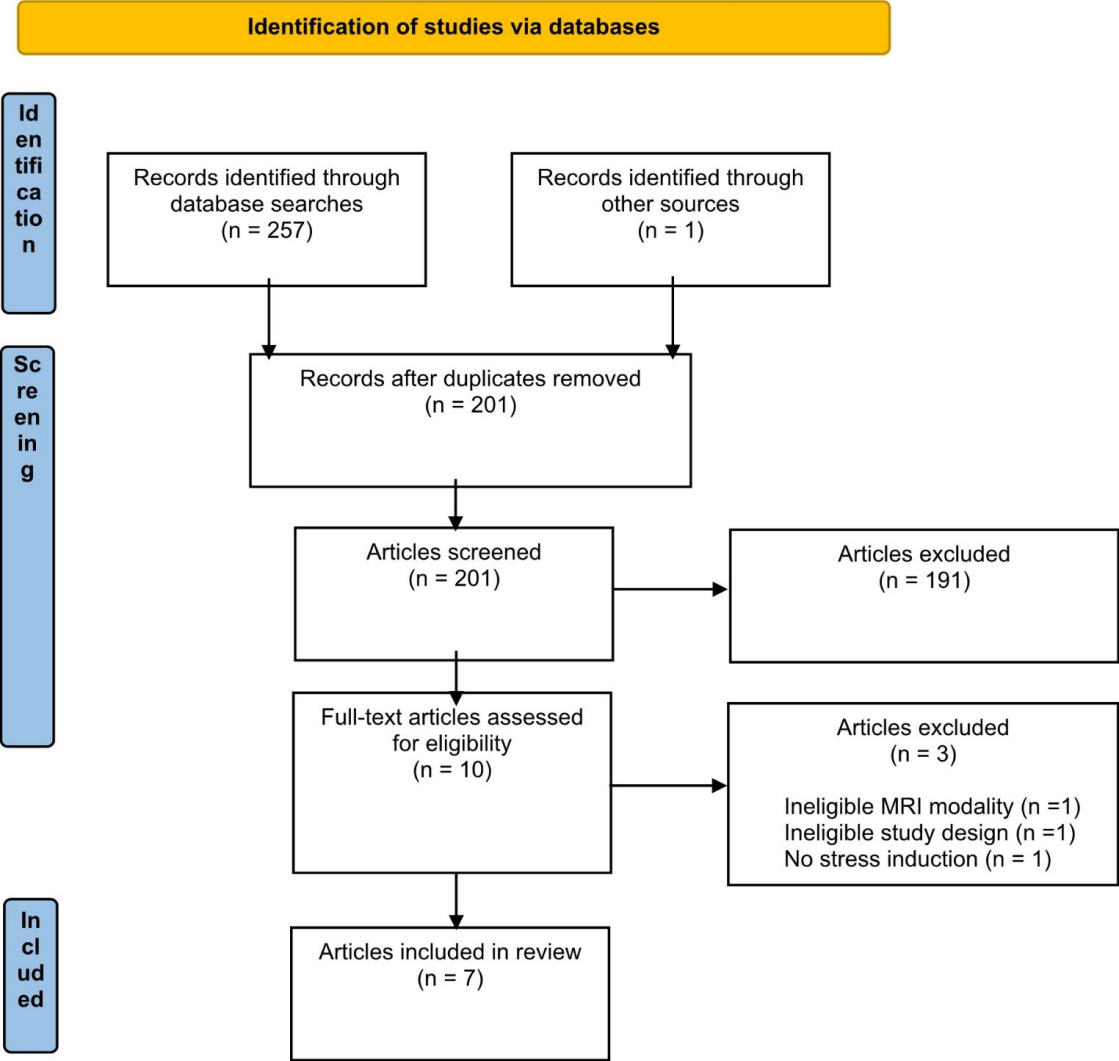
PRISMA flow chart of article selection process for the current review

Article references from initial database searches as well as those identified from reference list searches of eligible studies were uploaded into ‘Covidence’ (www.covidence.com), an online platform for article screening and data extraction. Duplicate publications were removed, leaving a total of 198 articles for screening. All articles were initially screened for eligibility based on their title and abstracts by two independent reviewers (E.G. and B.N.) Any discrepancies in screening outcomes resulted in a discussion between independent reviewers to determine the suitability of the article for the review. Discrepancies that could not be resolved through consensus were forwarded on to senior authors (A.V.G. and T.S.) for a final decision to be reached. The study selection process identified 7 articles that were included in the present review. Of these studies, an additional study was identified through backwards citation searches; however, it was not eligible for the current review. An updated literature search conducted on 01/05/2023 indicated an additional two eligible articles had since published since our original search. The study selection process is outlined in Fig. 1.

### Data extraction

A data extraction form was developed and piloted on three randomly selected studies by two independent reviewers (E.G. and B.N.). The following information was extracted from eligible articles: author, year of publication, country of publication, demographics (number of subjects, age, gender, classification of disinhibited eating), stress induction and stress measurement, results (including behavioural and key fMRI findings). Any articles that did not report required information resulted in one reviewer (E.G.) contacting the corresponding author for the relevant information.

### Quality assessment

Quality assessment for eligible studies was assessed using the Newcastle-Ottawa Scale [58], which rates the quality of non-randomized studies across three different domains: Selection, Comparability, and Outcome. Given the varying study formats across eligible studies, both the original cohort and the adapted cross-sectional format [59] were used where applicable. In the cohort format, a maximum of 9 points can be allocated across the three domains (Selection: 4; Comparability: 1; and Outcome: 3); and a maximum of 10 points can be allocated across domains in cross-sectional format (Selection: 5; Comparability:1; Outcome: 3). Higher scores are indicative of higher study quality. We considered scores ≥ 7 indicative of ‘good’ study quality, those between 2 and 6 points as ‘fair’, and those below 2 points as ‘poor’.

### Exploratory search

Given the limited number of eligible papers from our original search, we conducted a *post-hoc* exploratory search using broader selection criteria. Specifically, we included studies comprising participants who may experience DE in the presence of inappropriate compensatory behaviours (i.e., BN, AN-BP) as well as other clinical diagnoses that may also experience heightened levels of DE under certain conditions (i.e., night-eating syndrome). The purpose of this search was to (1) scope the size and characteristics of literature examining the neural correlates of stress on DE across a broader DE population; and (2) explore whether results from our initial search similarly captured findings from a broader DE population.

The search was conducted on 01/05/2023, with specific search terms available in Supplementary Table 2. Our search strategy, data selection, and data extraction processes remained identical to those used for the original search. The only modification was adjusting the inclusion criteria for the DE group to include participants with purging behaviours and night-eating syndrome; and the exclusion criteria was adapted to exclude participants with exclusively purging behaviours (i.e., purging disorder). The PRISMA flow-chart outlining article selection process for this exploratory search can be found in Supplementary Fig. 1.

## Results

### Study characteristics

Participant characteristics, methodological design, and main findings of each study are summarised in Table 2.

**Table 2:** Summary of study characteristics and main findings

**Reference**	**Country**	**Sample**	**Disinhibited eating measure**	**Stressor**	**Functional task**	**Stress measurement**	**Variables controlled for**	**Main behavioural findings**	**Main fMRI findings**
Carnell et al. (2022) [63]. *	United States.	17 adults with obesity: 6 with binge-eating symptoms (Age: *M* = 36.0 *SD* = 10.4; BMI: *M* = 36.0, *SD* = 3.0) and 11 without (Age: *M* = 35.6 *SD* = 7.3; BMI: *M* = 34.6, *SD* = 4.0), and 12 healthy weight controls (Age: *M* = 32.5 *SD* = 8.8; BMI: *M* = 22.6, *SD* = 1.3).	QEWP, EDE.	SECPT	Food word reactivity task.	Self-report (VAS), salivary cortisol.	BMI, sex, hunger.	Individuals with obesity, but without binge-eating symptoms, consumed more calories following stress induction in and *ad libitum* meal. However, both baseline and post stress caloric consumption was greater in those with binge-eating versus those without.	Under acute stress, higher- weight individuals with binge-eating demonstrated greater activation of the thalamus, sPFC, cerebellum and cuneus, and deactivation of the MTG in response to high calorie food words, relative to higher weight individuals without binge-eating. Under stressful conditions, food words relative to neutral words were associated with greater activation of OFC, superior frontal cortex, putamen, MTG, ACC, PCC, superior frontal cortex, and deactivation of the ilPFC in higher weight individuals with binge-eating relative to higher weight controls.
Chang et al. (2022) [64].	United States.	13 adults with emotional eating (Age: *M* = 28.6, *SD* = 5.8; BMI: *M* = 26.2, *SD* = 3.4), and 15 without emotional eating (Age: *M* = 28.0, *SD* = 5.4; BMI: *M* = 25.5, *SD* = 5.8).	DEBQ.	MAST.	FID task.	Self-report (VAS), blood serum cortisol.	BMI, education, sex, hunger.	No significant difference in *ad libitum* snack consumption between emotional and non-emotional eaters.	Acute stress was associated with reduced activity in the caudate, NAc, and putamen during anticipation of reward in those with emotional eating relative to those without emotional eating. No significant differences were observed between participants with and without emotional eating during stress or at baseline during reward outcome.
Hartogsveld et al. (2022) [65]. *	The Netherlands.	38 adults with binge-eating (Age: *M* = 26.11, *SD* = 6.49; BMI: *M* = 25.50, *SD* = 4.88), and 38 healthy controls (Age: *M* = 23.84, *SD* = 4.25; BMI: *M* = 23.23, *SD* = 2.55).	EDE.	MAST.	Adapted Slips-of-Action task.	Self-report (VAS), salivary cortisol, blood pressure.	Age, sex, BMI, prior stress test exposure.	All participants were more likely to select stimuli with valued outcomes (i.e., newly reinforced) over devalued (i.e., previously reinforced) outcomes regardless of stress exposure and binge-eating status.	Healthy controls under neutral conditions demonstrated activation of the ACC, OFC and insula in response to valued versus devalued outcomes. Activation did not significantly differ under stressful conditions in healthy controls. Under neutral and stressful conditions, individuals with binge-eating demonstrated less activation of the posterior putamen in response to valued versus devalued outcomes relative to those without binge-eating.
Jarcho et al. (2015) [66].	United States.	10 adolescents with LOC over eating (Age: *M* = 15.4, *SD* = 1.7; BMI: *M* = 33.5 *SD* = 9.2), and 12 without LOC over eating (Age: *M* = 16.1, *SD* = 1.4; BMI: *M* = 32.4 *SD* = 5.1).	EDE.	Chatroom task.	*As previous.*	Self-report (VAS).	BMI, age, hunger.	No significant difference in ad libitum snack consumption in adolescents with LOC and without LOC of eating.	Both LOC and no LOC groups displayed increased vmPFC activity in response to positive peer feedback. The LOC group displayed reduced activity in the vmPFC and dlPFC in response to negative peer feedback relative to no LOC. Negative peer feedback was associated with reduced vmPFC-striatum connectivity in LOC, but increased connectivity in no LOC. FFA activity was positively associated with *ad libitum* snack intake in those with LOC only.
Lyu & Jackson (2016) [28].	China.	18 adults with BED symptoms; 9 allocated to an acute stress exposure (Age: *M* = 19.22, *SD* = 0.44; BMI: *M* = 20.80, *SD* = 1.48) and 9 allocated to a control task (Age: *M* = 19.89, *SD* = 1.54; BMI: *M* = 20.72, *SD* = 2.34). 26 healthy controls; 12 allocated to an acute stress exposure (Age: *M* = 20.00, *SD* = 1.41; BMI: *M* = 19.19, *SD* = 1.52) and 14 allocated to a control task (Age: *M* = 19.43, *SD* = 1.34; BMI: *M* = 19.22, *SD* = 2.16).	EDDS, UES.	CPT with performance feedback	Passive observation of high-calorie, low-calorie and control stimuli.	Self-report (VAS).	BMI, age, hunger, psychiatric and neurological illness.	Acute stress was associated with lower palatability ratings for high-calorie foods in the low BED-symptomatic relative to high BED-symptomatic group. High BED-symptomatic participants in the stress condition are significantly more chocolate than low-BED symptomatic in the stress and control condition, but only ate slightly more than high BED-symptomatic participants in the control condition.	Acute stress was associated with reduced activation of the hippocampus, IFG, amygdala and insula in high BED-symptomatic relative to the low-BED group during high calorie > low calorie contrast. The control condition was associated with reduced activity in the ACC and SFG, and increased activity in the left OFC and putamen in high BED-symptomatic relative to low BED-symptomatic participants. Reduced hippocampal activity was positively correlated with increased chocolate consumption following the functional scan.
Tryon et al. (2013) [68].	United States.	16 adults with high chronic stress (Age: *M* = 46.81, *SD* = 1.09; BMI: *M* = 26.76, *SD* = 1.35) and 14 adults with low chronic stress (Age: *M* = 31.64, *SD* = 3.64; BMI: *M* = 24.27, *SD* = 1.26).	*Ad libitum* food consumption following acute stress induction using the TSST.	Chronic, life stress	*As above.*	Self-report (VAS, WCSS), salivary cortisol.	*As above.*	High chronic stress was associated with significantly greater *ad libitum* snack consumption following acute stress induction. The high chronic stress group displayed blunted cortisol reactivity relative to low chronic stress group following acute stress induction, and blunted reactivity at waking and midday.	In the high calorie > control contrast, high relative to low chronic stress was associated with greater activation of the ACC, anterior amygdala, right mOBC, left putamen and caudate, and reduced activation in the contralateral caudate and putamen, bilateral dlPFC, left anterior PFC, right ACC, and left lOBC. High calorie > low calorie contrasts were associated with increased bilateral activation in the amygdala, right ACC, left putamen and right mOBC, and reduced activity of the left anterior PFC and dlPFC in high relative to low chronic stress.
Wagner et al. (2012) [67].	United States.	30 adult chronic dieters (Age: *M* = 19.7; BMI: *M* = 21.4).	Restraint Scale	VMIP	Food-cue reactivity task	Self-report (VAS)	BMI, age, neurological illness.	Negative relative to neutral mood induction was associated with significantly reduced self-esteem.	Negative relative to neutral mood induction was associated with greater OBC activity response to palatable food cues. OFC and NAc activity in response to palatable food cues was negatively correlated with the degree of self-report self-esteem change following negative mood induction.

*Note.* *, denotes studies included after the exploratory search; ACC, Anterior cingulate cortex; BED, binge-eating disorder; BMI, body mass index; CPT, Cold Pressor Test; DEBQ, Dutch Eating Behaviour Questionnaire; dlPFC, dorsolateral prefrontal cortex; EDE, Eating Disorder Examination; EDDE, Eating Disorder Diagnostic Examination; FFA, fusiform ‘face’ area; FID, Food Incentive Delay; IFG, inferior frontal gyrus; ilPFC, inferior lateral prefrontal cortex; LOC, loss of control; lOFC, lateral orbitofrontal cortex; M, mean; MAST, Maastricht Acute Stress Test; mOFC, medial orbitofrontal cortex; MTG, middle temporal gyrus; NA: not applicable; NAc: nucleus accumbens; OFC: orbitofrontal cortex; PCC, posterior cingulate cortex; cortex; QEWP, Questionnaire on Eating and Weight Patterns; SECPT, Socially Evaluated Cold Pressor Test; SFG, superior frontal gyrus; SD, standard deviation; sPFC, subgenual prefrontal cortex; TSST, Trier Social Stress Test; UES, Uncontrolled Eating Scale; VAS, visual analogue scale; VMIP, Velton Mood Induction Procedure; vmPFC, ventromedial prefrontal cortex; WCSS, Wheaton Chronic Stress Scale.

The seven eligible studies yielded data from 259 participants, with sample sizes ranging from 22 to 76 (*M* = 20.54, *SD* = 15.26). Most studies had female only samples, except for Carnell et al. (55% female) [63], Chang et al. [64] (50% female), and Hartogsveld et al. [65] (80% female). The pooled mean age of the samples was 26.38 years (DE *M* = 26.26, controls *M* = 26.28), and the pooled average BMI was 25.98 kg/m^2^ (range = 20.72-36.00 kg/m^2^ ) in participants with DE and 25.85 kg/m^2^ (range = 19.19–34.6 kg/m^2^ ) in controls. Only one study included a paediatric sample (< 18 years old) [66].

Six studies assessed DE using self-report measures or clinical interviews [28, 63, 64, 66, 67]. Alternatively, Tryon et al. [68] classified participants as presenting with either ‘high’ or ‘low’ chronic stress based on median scores in the Wheaton Chronic Stress Scale (WCSS) [69]. Following this, acute stress was induced in participants and *ad libitum* snack intake was used as a behavioural proxy of DE.

In relation to studies assessing the effects of acute stress, there was notable heterogeneity in paradigms used across studies. Four studies [28, 63–65] used stress-induction protocols that involved combinations of mild pain and social evaluation stressors (Cold Pressor Test, [70] with negative performance feedback; socially evaluated CPT, [63]; Maastricht Acute Stress Test, [65, 71]). Wagner et al. [67] used a negative emotion task (Velten Mood Induction Procedure, [72]), which involves inducing negative mood via 60 negative, self-referential statements. Alternatively, Jarcho et al. [66] used a social evaluation / threat task (Chatroom Task, [73, 74]; described below) to induce stress. It should be noted that, although Tryon et al. [68] did use the Trier Social Stress Test (TSST) [75] as a method of acute stress induction in their study, this was not directly related to any fMRI analyses. Therefore, we do not include this an acute stress induction paradigm in the current review. Only one study, Tryon et al. [68], assessed levels of chronic stress in participants. As mentioned, chronic stress was assessed using the WCSS [62], which assesses the presence and severity of common chronic stressful life events related to work, relationships, and financial strain across 51 items.

Each study assessed changes in subjective stress levels in participants using self-report, and four studies measured stress-related changes in cortisol secretion. Of the studies that assessed cortisol levels, three [63, 65, 76] assessed salivary or blood serum cortisol before and after acute stress exposure, while one [68] assessed diurnal cortisol levels over a 24-hour period.

Five studies used fMRI paradigms that assessed food-related reward-sensitivity. Chang et al. [64] used a Food Incentive Delay (FID) task that assesses both anticipatory and consummatory phases of reward-processing. In the FID, participants are either presented with a neutral stimulus indicating either reward (food) or a neutral (i.e., random shape) outcome prior to a short, variable delay (anticipation phase). Following this, participants must respond to a target within a brief, pre-determined time frame. Notification of response success results in presentation of the anticipated cue (i.e., food or random shape) with a tick on top, whereas response failure will result in an X (outcome phase). Wagner et al. [67] used a food-cue reactivity task, which requires participants to respond via button press to various stimuli, including high-calorie foods, people, and natural scenery. Participants are required to respond to any stimuli taking place indoors to conceal the true purpose of the task (i.e., reactivity to food stimuli). Carnell et al. [63] used a food word reactivity task, which involved the presentation of words related to palatable, high calorie foods as well as neutral words. Alternatively, Lyu & Jackson [28] and Tryon et al. [68] used paradigms involving presentation of high-calorie, low-calorie, or control stimuli (i.e., familiar, emotionally neutral, non-food objects) with no response component.

Alternatively, Jarcho et al. [66] used a social evaluation / threat fMRI paradigm, the Chatroom Task. This task assesses neural responses to perceived social acceptance or rejection. Participants are required to rate images of virtual peers depending on whether they would be interested (high-value) or not interested (low-value) in talking to them in a virtual chatroom prior to the functional scan. During the scan, participants are informed virtual peers have made reciprocal ratings, and they must determine whether how they had been rated by these peers, and how much they had expected their peers’ ratings. Hartogsveld et al. [65] used an adapted version of the Slips of Action task, in conjunction with an instrumental learning and devaluation protocol that occurred prior to the fMRI paradigm. In brief, participants were required to learn various stimulus-response- outcome (SRO) associations for chocolate and potato chips, of which a correct button press (left or right key) would result in reward 75% of the time. These associations were devalued by allowing participants to select and consume either chocolate or chips to satiety. This was followed by the SOA task, participants must respond to chocolate or chip stimuli, however button responses for either stimulus were reversed. Slips occurred when participants selected foods that had been devalued, but selection of valued foods were considered correct responses.

As the current review yielded a small number of studies with heterogeneous and complex designs, we have described the results in relation to key design features to facilitate greater understanding of key findings across studies. Furthermore, we have also described the findings in more detail than generally seen in reviews involving a larger pool of studies.

### Neuroimaging findings

#### Changes in neural activity associated with acute versus chronic stress

Acute stress paradigms employing both mild pain and social evaluation were associated with significant deactivation of mesocorticolimbic regions in response to high-calorie food cues in those with DE relative to controls. However, these specific regions varied across studies. For example, Lyu & Jackson [25] identified acute stress was associated with deactivation of the hippocampus, insula and IFG in the DE group when viewing high-calorie relative to low-calorie stimuli. However, Chang et al. [53] observed that the DE group displayed heightened deactivation of the caudate and putamen under stressful conditions when viewing high-calorie stimuli.

Of the studies using acute stress paradigms with only psychosocial elements, stress was associated with significantly greater activation of frontal regions in those with DE. Wagner et al. [61] identified that this activation was localised in the orbitofrontal cortex when viewing high-calorie relative to low-calorie foods. However, Jarcho et al. [66] had more nuanced findings, with all participants displaying heightened ventromedial prefrontal cortex activation in response to positive peer feedback during the Chatroom task, while only those in the DE group displayed significant deactivation of the medial dorsomedial prefrontal cortex in response to negative feedback (stress).

Alternatively, Tyron et al. [63]’s study, which considered the influence of cumulative stress in response to high-calorie relative to low-calorie images, elevated chronic stress was associated with greater activation in the amygdala, the left putamen and caudate, and the ACC and right medial OFC. In addition, elevated chronic stress was linked to greater deactivation in the contralateral (right) side of the caudate and putamen, bilaterally in the dlPFC and in the left anterior prefrontal cortex, and the left lateral OFC. Relative to low-calorie images, high-calorie images were associated with greater activation in the left putamen, right medial OFC and ACC, and bilateral amygdala and caudate in the high chronic stress group relative to low chronic stress.

#### Impact of food vs. non-food cues on DE-related fMRI findings

Five of the seven included studies used tasks that investigated reward-processing of food cues. In relation to those that assessed active responding, there were significant interactions of group (i.e., DE) and session (i.e., stress versus neutral mood) within mesocoritcolimbic regions implicated in reward processing, including the nucleus accumbens, caudate, putamen and OFC. However, the nucleus accumbens was only common region to be significantly activated in response under stressful conditions in the DE group relative to controls. Furthermore, the direction of differences in nucleus accumbens activation differed across studies. While Chang et al. [64] identified diminished recruitment of the nucleus accumbens in those characterised by DE, Wagner et al. [67] identified greater negative mood during stress induction was associated with heighted nucleus accumbens recruitment when viewing high-calorie food cues. These differences may be attributable to task differences, specifically Chang et al. [64] only observed significant nucleus accumbens deactivation during the anticipatory phase of high-calorie food-cue presentation, whereas Wagner et al. [67] observed nucleus accumbens activation during reward receipt.

Of the two studies that assessed neural activation in response to food-cues alone (i.e., no response element), both identified that the DE group displayed significant changes in anterior cingulate cortex and amygdala in response to high-calorie food-cues when stressed. However, while Lyu and Jackson [28] identified stress was associated with diminished activity in the amygdala and anterior cingulate cortex in when viewing high-calorie food cues in the DE group relative to controls, Tryon et al. [68] identified the opposite effect. However, considering these studies assessed acute and chronic stress, respectively, it is plausible these differences can be attributed to stress duration.

Only one study [65] examined the effects of stress on goal-directed and habitual learning of food-cue associations. Acute stress was associated with diminished recruitment of the putamen between trials that assessed goal-directed versus habitual behaviour in those with DE relative to healthy controls. However, other regions typically implicated in mediating goal-directed behaviour, such as the orbitofrontal cortex and anterior cingulate cortex were not significantly affected by stress in those with DE.

### Behavioural findings

Five studies assessed *ad libitum* food consumption after acute stress induction [28, 63, 64, 66, 68]. Three studies observed changes in food consumption following acute stress induction. Lyu & Jackson [28] observed that those with DE ate significantly more chocolate than those without DE under acutely stressful conditions. Furthermore, chocolate consumption was significantly negatively correlated with hippocampal deactivation in high calorie versus low calorie condition. Similarly, Tryon et al. [68] observed that participants with high cumulative stress exposure ate significantly more high-calorie foods relative to those who had not experienced as much cumulative stress following acute stress exposure. Carnell et al. [63] also observed differences in *ad libitum* food intake among higher weight adults with DE versus those higher weight adults without DE, however those with DE were found to decrease intake following acute stress induction, while those without ate more than at baseline. Regardless, those with DE ate more at baseline and post-stress relative to their counterparts.

Although Jarcho et al. [66] observed no differences in the quantity of *ad libitum* food consumption between those with and without disinhibited eating traits, activity in the fusiform ‘face’ area during negative feedback trials was positively associated with overall food intake in the disinhibited eating group, but not in controls.

### Quality assessment

The results of the quality assessment for eligible studies are summarised in Table 3. The quality of studies ranged from fair to good. The main areas for improvement include Selection (i.e., the quality of DE classification and selection of appropriate controls) and Comparability (i.e., controlling for appropriate confounding variables). Most studies performed well on the Outcome domain.

**Table 3:** Quality assessment outcomes for eligible studies

Reference	Selection	Comparability	Outcome	Overall score
**Cross-Sectional**				
Jarcho et al. (2015).	+ + +	+ +	+ + +	Good
Tryon et al. (2013).	+ +	+ +	+ + +	Good
**Cohort**				
Carnell et al. (2022).	+ +	+ +	+ + +	Good
Chang et al. (2022).	+ +	+ +	+ + +	Good
Hartogsvled et al. (2022).	+ + +	+ +	+ + +	Good
Lyu & Jackson (2016).	+	+ +	+ + +	Fair
Wagner et al. (2012).	+ +	+ +	+ +	Fair

*Note.* + indicates 1 point. Quality was assessed using the Newcastle-Ottawa scale. For studies using the cross-sectional format, a maximum of 10 can be allocated (Selection: 5; Comparability:2; Outcome: 3). For studies assessed using the cohort format, a maximum of 9 points can be allocated (Selection: 4; Comparability: 2; and Outcome: 3). We considered scores ≥ 7 indicative of ‘good’ study quality, those between 2–6 points as ‘fair’, and those below 2 points as ‘poor’.

### Exploratory literature search

The exploratory literature search identified five eligible articles. These articles yielded data from 190 participants, of whom 98 had symptoms of BN, 22 had symptoms of AN-BP, and 70 were healthy matched controls. Specific details about participant characteristics and study methodology can be found in Supplementary Table 3.

Four of these studies induced acute stress using paradigms some element of challenging mental load (i.e., difficult arithmetic tasks) and social evaluation [77–79], while one used an affect-induction paradigm [80].

In relation to fMRI protocols, Collins et al. [77], Fischer et al., [78] and Wonderlich et al. [81] used food-cue reactivity tasks. Overall, individuals with BN demonstrated heightened activation of the cuneus, and diminished recruitment of limbic (i.e., amygdala) and prefrontal regions (ventromedial and dorsolateral prefrontal cortices), as well as the anterior cingulate cortex in response to palatable / high-calorie food-cues under acute stress relative to healthy controls [77, 78, 81]. Moreover, diminished activation in the ventromedial prefrontal cortex was modulated by heightened self-reported stress prior to binge-eating in those with BN [78, 81].

Westwater et al. [79] used the Stop Signal Anticipation Task (SSAT) to assess reactive and proactive inhibition in individuals with BN and AN-BP. In brief, the SSAT requires participants to respond to specific stimuli (go-signal) via button-response, while withholding responses to other stimuli that occur shortly after Go-signal presentation (stop-signal). The probability of stop-signal occurrence varies across trials and is indicated by a cue (i.e., colour). Proactive inhibition is measured as the ability to successfully slow reaction time when a cue indicating high stop-signal probability is present. Alternatively reactive inhibition is measured as the latency between stop-signal presentation and response inhibition. Individuals with BN had difficulty with proactive inhibition relative to those with AN-BP and control participants, but performance was not modulated by stress exposure. However, when acutely stressed, proactive inhibition was associated with greater recruitment of the superior frontal gyrus in those with BN relative to AN-BP and controls. Both those with BN and AN-BP demonstrated diminished recruitment of the ventromedial prefrontal cortex during proactive inhibition when stressed.

Finally, Dreyfuss et al. [80] used an emotional go/no-go fMRI paradigm which requires participants to respond only to faces displaying specific emotions (i.e., happy) while withholding responses to other emotions (i.e., fearful) or neutral faces. There were no significant differences in response accuracy to faces displaying negative or neutral affect between individuals with BN and healthy controls, however those with BN demonstrated greater response accuracy to positive affect. Healthy controls demonstrated heightened activation of the middle frontal gyrus and subgenual cingulate during successful trials, however this was not observed in those with BN. Moreover, age predicted greater recruitment of these regions during successful trials in healthy controls, but not those with BN.

## Discussion

This review systematically analysed literature examining the neural correlates of stress and DE in order to identify emerging patterns in existing work and to suggest improvements for future research. Given the small number of studies included in the review, coupled with the heterogeneity of study designs, we offer cautious interpretations of our findings.

### Acute stress

#### The impact of acute stress on reward circuitry

We hypothesised that acute stress would be associated increased recruitment of the mesocorticolimbic reward network in people with DE. However, the identified effects of acute stress on mesocorticolimbic circuitry underlying reward-processing in DE were mixed. While studies by Chang et al. [64] and Lyu and Jackson [28], and Carnell [63] suggest that DE was associated with diminished recruitment of subcortical reward regions (i.e., nucleus accumbens, caudate, putamen) and the orbitofrontal cortex under acute stress, there was also contrary evidence to suggest acute stress may enhance recruitment of the nucleus accumbens and thalamus with DE [65, 67]. These mixed findings are somewhat unexpected, provided heightened activity within both subcortical reward regions frequently coincides with increased preference for food-rewards in individuals with BED [51, 82], emotional eating [83, 84], and in young people with DE [85]. Indeed, sensitisation of the mesocorticolimbic network has been widely regarded as a key mechanism of DE [1, 52], namely driving the increased incentive-motivation (i.e., wanting/craving) for energy-dense, palatable foods. Further to this, acute stress has been associated with increased activity within ventral striatum (nucleus accumbens), caudate, and orbitofrontal cortex during monetary and food reward anticipation [61, 86], as well as increased connectivity between the ventral striatum and ventromedial prefrontal cortex (which is implicated in integrating sensory input and reward value) when selecting energy-dense foods during food choice [86].

There are several possible explanations for these findings. Although hyperactivity of reward regions has often been observed in those with DE, some evidence suggests hypoactivity of the mesocorticlimbic circuit may also occur. For example, Balodis et al. [87] observed diminished recruitment of the ventral striatum during anticipation of monetary rewards, and attenuation of the medial prefrontal cortex during receipt, in individuals with BED. Similarly, another study by Simon et al. [88] found that individuals with binge-eating spectrum disorders display diminished recruitment of the posterior cingulate cortex when anticipating food reward, and increased activation of the posterior cingulate cortex and anterior medial prefrontal cortex during receipt. These findings align with an alternative suggested mechanism for DE, wherein repeated consumption of energy-dense foods may eventually desensitise reward circuitry [89]. In turn, DE behaviours may continue as a means of overcoming diminished mesocorticolimbic responsivity to such foods. Therefore, it is a possibility that acute stress does not drive DE through increasing recruitment of reward regions as we predicted, but rather may exacerbate hyper-responsivity of the reward circuitry to energy-dense and palatable food cues. There is evidence to suggest acute stress decreases activation of the putamen, amygdala and orbitofrontal cortex when selecting unhealthy foods post-prandially in healthy adults [90]. However, whether this finding would extend to populations characterised by DE would require further investigation.

Another reason for these unexpected findings could be attributed to stress induction paradigms. While Chang et al. [64], Lyu and Jackson [28], and Carnell et al. [63] used procedures designed to induce stress, Wagner et al. [67] used the VMIP, which was originally designed as a negative mood induction procedure. Although negative mood is often heightened following stress induction procedures [71, 91, 92], and correspondingly the VMIP has previously demonstrated significant elevations of cortisol [93], it is possible that use of self-referential statements (i.e., internally generated negative affect) in the VMIP may have been more effective in triggering negative affect than the Cold Pressor Test or Maastricht Acute Stress Test [94]. Previous behavioural evidence suggests that higher levels of negative affect during acute stress are stronger predictors of stress-induced eating [95]. Moreover, heightened levels of negative affect in women with BN has been associated with greater activity in the caudate, putamen and pallidum during food reward receipt [96]. Taken together, it is possible that the negative affectivity component of stress may be more effective in driving incentive-motivation for rewards under stress in DE. Indeed, this would align with affective-regulation models of DE, which suggest overeating palatable, energy-dense serves to negate negative mood states induced by stress [35–37]. Overall, the effects of acute stress on the neural correlates of reward processing in DE currently remain unclear, with conflicting results likely a result of inconsistencies in study design.

#### The influence of acute stress on cognitive control circuitry

We also hypothesised that acute stress would be associated with decreased recruitment of prefrontal regions implicated in cognitive control in people with DE. In support of this hypothesis, there was evidence to suggest acute stress increases aberrant recruitment of regions implicated in cognitive control. For example, Lyu and Jackson [28] identified acute stress relative to a less stressful condition was with diminished recruitment of the inferior frontal gyrus when viewing high calorie food stimuli in BED-symptomatic women relative to controls. The inferior frontal gyrus has been consistently implicated in response inhibition [97, 98]. Previous research has identified that people with BED, and emotional eating, display diminished recruitment of the inferior frontal gyrus during attempts to inhibit prepotent motor responses, and also during the anticipation of food-reward [87]. Consequently, it has been speculated that aberrant recruitment inhibitory control regions, such as the inferior frontal gyrus, may underpin subjective loss of control characteristic of DE. Stress-induced attenuation of the inferior frontal gyrus in response to high calorie food cues were also accompanied by significant differences in *ad libitum* food consumption between groups, with the BED-symptomatic group consuming significantly more food post-stress manipulation relative to controls. Similarly, Carnell et al. [63] observed acute stress was associated with deactivation of the inferior lateral prefrontal cortex in individuals with DE when viewing food versus neutral words. The inferior lateral prefrontal cortex has also been implicated in inhibition of inappropriate prepotent responses, with activation in this region known to predict individual differences in inhibitory control performance [99]. Taken together, these findings suggest that acute stress may exacerbate DE symptoms, in part, via impairing inhibitory control processes.

Furthermore, Jarcho et al. [66] identified that young people with loss-of-control eating display diminished recruitment of the dorsolateral prefrontal cortex and ventromedial prefrontal cortex relative to healthy controls when experiencing acute psychosocial stress (negative peer feedback). Diminished recruitment of the ventromedial prefrontal cortex has previously been associated with heightened levels of negative affect in healthy adult and paediatric samples [100–102], as well as poorer ability to downregulate negative emotions in women with obesity relative to healthy weight controls [57]. Similarly, increased activation of the dorsolateral prefrontal cortex has also been associated with successful downregulation of negative affect elicited by social exclusion [103]. Therefore, attenuation of both the ventromedial and dorsolateral prefrontal cortex during acute stress social stress in young people with loss-of-control eating may reflect diminished ability to downregulate negative emotions elicited by negative peer feedback. Although activity in both the ventromedial and dorsolateral prefrontal cortices did not distinguish *ad libitum* food consumption between those with and without loss-of-control eating, heightened activation in the fusiform “face” area was associated with greater food consumption in those with loss-of-control eating. The fusiform “face” area has previously been shown to respond to social exclusion during social interaction paradigms [104]. As such, the subjective experience of social exclusion in young people with loss-of-control eating may be a salient antecedent for DE episodes, especially if accompanied by impaired ability to sufficiently regulate negative emotions.

Collectively, existing literature supports our hypothesis that impaired recruitment of brain regions involved in cognitive control processes, specifically those related to inhibitory control and affect regulation, may contribute to increased DE under acute stress. These findings provide further support for affective-regulation models of DE, wherein negative emotional states may increase the likelihood of impulsive responding, as well as increased attempts to alleviate negative affect via external rewards (i.e., food) as internal methods of coping may not be sufficient [36].

#### The influence of acute stress on interoception circuitry

Our third hypothesis predicted that acute stress would be associated with increased recruitment of regions of the salience network in people with DE. In alignment with our hypothesis, Carnell et al. [63] identified that individuals with DE and higher body weight demonstrated greater activation of the anterior cingulate cortex in response to viewing high calorie versus neutral words under acute stress. The anterior cingulate cortex has been implicated in several roles, including attentional allocation and reward-based motivation [105, 106]. Therefore, in the context of this study, stress-induced anterior cingulate cortex activation may reflect increased motivational salience towards high calorie food cues. However, it should be noted that this result was classified as exploratory in the original sample, given the relatively small number of individuals with DE. Therefore, whether this effect would persist with a larger sample is uncertain.

Lyu and Jackson [28] identified that acute stress was associated with significantly diminished recruitment of the hippocampus in BED-symptomatic women relative to controls. Moreover, greater hippocampal attenuation was significantly associated with *ad libitum* food consumption in the BED-symptomatic women. Although the hippocampus has long been considered and integrative hub for visuo-spatial and memory processing, there is evidence to suggest it may also play a role in energy homeostasis via interoceptive cues [107]. For example, receptors for endocrine hormones signalling satiety (i.e., leptin) and hunger (i.e., ghrelin) have been found within the hippocampus [108, 109]. Ghrelin has also been identified to interact with the ventral hippocampus to elicit meal-conditioned appetite, or in other words the stimulation of appetite via Pavolvian reinforcement of environmental antecedents of meal consumption [110]. Moreover, bilateral lesions of the hippocampus in humans have been associated with elevated levels of subjective hunger after meal consumption, suggesting the hippocampus plays a direct role in regulating interoceptive feelings of hunger and satiety. Impairments in interoceptive processing of satiety and emotional states have also been identified in populations characterised by DE, including individuals with BED [111] and emotional eating [112, 113]. While it is plausible that, given the hippocampus’ role in interoception and homeostasis, increased *ad libitum* food consumption may have been attributable to impaired interoception, Lyu and Jackson’s study design does not sufficiently permit for such conclusions. Consequently, future work may endeavour to further investigate the impact of stress on the hippocampus in DE, and its potential role influence interoception, using emerging statistical methodologies, such as computational modelling [114].

### Chronic stress

Only one study included in our review (Tryon et al. [68]) assessed the effects of chronic stress on DE. Our first hypothesis predicted that stress would increase recruitment of regions implicated in reward processing in people with DE relative to healthy controls. In support of this hypothesis chronic stress exposure was associated with heightened activity in subcortical reward processing (i.e., amygdala, caudate, putamen) in response to high-calorie food stimuli. These results were expected, given chronic stress exposure has been demonstrated to sensitise reward circuitry involved in food choice [115, 116], while also increasing neural plasticity in areas involved in stress reactivity, such as the amygdala and anterior cingulate cortex [115, 117]. Provided DE is generally characterised by heightened sensitivity of mesocorticolimbic reward circuitry [118, 119], it was also expected that chronically stressed individuals presented significantly greater activation within this circuit when viewing palatable food-cues relative to controls. These findings also consistent with heuristic models of chronic stress and obesity, which suggest that individual differences, such as disordered eating patterns, in conjunction with repeated chronic stress exposure hyperactivate dopaminergic signalling in striatal and limbic regions [120]. Overall, these findings lend support to the framework that chronic stress exposure may exacerbate already heightened reward sensitivity to palatable food cues in DE [121]. Further to this, high chronic stress was also associated with increased activation of the medial orbitofrontal cortex in response to high-calorie food-cues. The medial orbitofrontal cortex is involved in the representation of food reward and encoding the subjective pleasantness of food-cues [122]. In the context of Tryon et al.’s study, heightened medial orbitofrontal cortex activation in those with chronic stress exposure could reflect alterations in the subjective reward value attributed to high-calorie foods. This finding aligns with broader research indicating chronic stress biases food preference towards foods that are energy-dense foods, especially in individuals with higher weight and DE.

Our second hypothesis predicted that stress would decrease recruitment of prefrontal regions implicated in cognitive control. Consistent with this hypothesis, Tryon et al. [68] also found that high chronic stress correlated with significantly lower activation in the anterior and dorsolateral prefrontal cortex cortices relative to lower levels of stress. This is consistent with existing findings that suggest chronic stress may impair top-down cognitive processes involved in decision-making and executive functioning [123–125], with notably greater impairment often observed in higher-weight populations [126–129]. The anterior prefrontal cortex facilitates higher order cognition, such as planning and integrating the outcomes of other cognitive processes to achieve a higher order behavioural goal [130, 131]. Together with hypoactivation of the dorsolateral prefrontal cortex this pattern of neural activation is also consistent with the idea that repeated stress exposure may impair the ability to regulate emotional responses to palatable foods, while also impairing the ability to inhibit prepotent responses. Also coupled with increased sensitivity of reward and limbic circuitry, hypoactivation of anterior and dorsolateral prefrontal cortices in those with high chronic stress exposure is consistent with theoretical models of stress and binge-eating, which posit that while overconsumption of palatable foods when stressed may initially be an impulsive, goal-driven behaviour (i.e., to alleviate negative mood states), repeated pairing of stress and binging may eventually cause it to become a compulsive response [132, 133].

In relation to our third hypothesis that predicted stress may decrease activation of neural regions involved in interoceptive processes, Tryon et al. did not find evidence to suggest chronic stress significantly influenced activation in regions involved in interoception. While there is limited research investigating the relationship between chronic stress and interoception on a neural level, behavioural and endocrinological research indicates that chronic stress may impair the signalling of satiety [116], including those with DE [33, 134]. Further work is needed to elucidate whether neural mechanisms involved in interoception may be impaired with chronic stress in this population.

Altogether, these findings support theoretical frameworks of DE, that suggest cumulative stress exposure likely exacerbates or contributes to aberrant reward-processing, self-regulation that contribute to DE [135]. However, it should be noted that these findings were based on a single study alone [68], and that DE was characterised by a proxy measure (i.e., chronic stress exposure). Regardless, given chronic stress has been associated with an increased likelihood of DE [5, 136], and is strongly associated with the maintenance of obesity [137, 138] and other eating disorders [139], these findings highlight the importance of further clarifying the role of cumulative stress exposure and the neural underpinnings of DE-related behaviours.

### Exploratory search findings: comparison of neural correlates of stress in DE with and without compensatory behaviours

To address the small sample of articles yielded from our initial search, we conducted an exploratory search including search terms that encapsulated clinical diagnoses, other than BED, that were characterised by DE (i.e., BN, AN-BP, and night eating syndrome). Overall, this search also yielded a small number of studies. Of these studies, the majority assessed neural correlates of acute stress in BN symptoms, with one study including an AN-BP subgroup. No studies were found that assessed neural correlates of night eating syndrome.

There was some evidence to suggest individuals with DE and compensatory behaviours also display aberrant recruitment of reward circuitry under acute stress. However, the regions implicated varied from those identified in our original review. For example, acute stress was associated with diminished recruitment of the amygdala and ventromedial prefrontal cortex in response to high calorie food cues in individuals with BN symptoms. Interestingly, reward regions often triggered by food-cue reactivity paradigms, such as the nucleus accumbens were not significantly affected by stress in this population. This was somewhat unexpected, provided BN is typically characterised by aberrant recruitment of subcortical reward regions, as well as the orbitofrontal cortex in response to palatable food cues [50]. Taken together, it is possible that BN may be characterised by a differential impairment of reward regions under stress, with a more pronounced limbic and prefrontal response, relative to individuals who experience DE without compensatory behaviours.

There was also some evidence to suggest that individuals with BN and AN-BP also demonstrate impaired recruitment of cognitive control regions. While individuals with DE alone showed significant impairments in inferior frontal gyrus activation under stress, Westwater et al. found greater superior frontal gyrus activation in BN participants, and ventromedial prefrontal cortex deactivation in AN-BP participants during inhibitory control task. While both the inferior frontal and superior frontal gyri are implicated in inhibitory control, differences we observed across populations are likely attributable to task demands. More specifically, the task employed by Westwater et al. [79] required the use of both proactive and reactive inhibition, while Lyu & Jackson’s task did not involve motor responding. Correspondingly, the superior frontal gyrus is implicated in successful proactive inhibition, while the inferior frontal gyrus is involved in reactive inhibition and integration of reward and cognitive signals during inhibitory control. Furthermore, heightened superior frontal gyrus activation in those with BN may also reflect a compensatory mechanism used in proactive inhibition processes under stress, especially provided task performance was not different from healthy controls under similar conditions. Interestingly the ventromedial prefrontal cortex is not typically involved in motor-response inhibitory control tasks, and as acknowledged by Westwater and colleagues [79], future investigation may be required to understand this finding from the AN-BP group. Regardless, it appears those with BN, AN-BP and DE without compensatory behaviour display general impairments in cognitive control circuitry under stress. Future research may extend these findings to characterise both reactive and proactive inhibitory control are similarly impaired by stress across a broader spectrum of DE.

Moreover, Dreyfuss et al. [80] found poorer recruitment of the middle frontal gyrus and subgenual gyrus gyrus (ventromedial prefrontal cortex) under stress was moderated by older age in people with BN when viewing images of faces showing negative emotion relative to healthy controls. These findings indicate that recruitment of cognitive control under stress regions may vary with age in BN, suggesting the illness duration and developmental stage may be important factors in explaining cognitive control capacity in those with BN. They also complement Jarcho et al. [66], wherein social exclusion was associated with diminished recruitment of the ventromedial prefrontal cortex. Together, this suggests BN be similarly characterised by general impairments in regulating negative affect generated by social cues, and in turn this may have an influence on binge-eating emergence [31, 78, 81].

Finally, there was also some evidence to suggest BN is associated impairments in salience network regions under stress. Specifically, acute stress was associated with diminished recruitment of the anterior cingulate cortex during a food-cue reactivity task [78, 81]. Stress-induced deactivation of the anterior cingulate cortex when viewing palatable food cues aligns with Carnell et al.’s [63] findings and suggest that stress influence increases motivation for palatable foods may also be present in BN.

Overall, DE with compensatory behaviour show some similarities in cognitive control and salience network activation under acute stress relative to DE alone, however limbic and prefrontal regions implicated in reward processing may be more more relevant in those with compensatory behaviours. Divergent findings may be attributable to task design, as well as small sample sizes. However, it highlights the need to better understand whether DE with or without compensatory behaviours may present different endophenotypes under acute stress, and the implications this has for diagnosis and treatment.

### Limitations and future directions for research

#### Sample size & sample heterogeneity

Given the small number of articles identified within the current review, which in themselves had relatively small sample sizes, interpretations should be cautious. Additionally, there was notable heterogeneity in the characteristics of the samples included in the review. There were different methods used for categorising DE. While most studies [28, 63–65, 67] opted to use clinically validated measures of binge-eating and unrestrained eating behaviours, others [68] used proxy measures of DE (i.e., chronic stress). Even among those that chose to use validated questionnaires or clinical interviews, studies often used different measures. Although DE is conceptualised as a spectrum of unrestrained eating behaviours that vary in severity [1, 140], the lack of consistent measurement across studies may introduce undesired bias or fail to capture essential elements of the construct. Consequently, this current gap in classification perhaps highlights the need to better standardise the construct of DE across varying levels of severity. Developing and committing to the use of a ‘gold-standard’ measure of DE may be a useful way of overcoming this limitation. Psychometric approaches, such as Item Response Theory, may be a useful way of harmonizing existing measures of questionnaires assessing DE. Indeed, Item Response Theory has previously used to identify common measures of eating disorder pathology converge on a single common construct that closely resembles DE [140].

#### Methodological heterogeneity

Of the studies that assessed acute stress, four studies [28, 63–65] used stress induction paradigms featuring both acute and physiological stressors (i.e., Maastricht Acute Stress Test, Cold Pressor Test with social evaluation), while the remainder [66, 67] did not include elements of physiological stress. It is plausible the inclusion of physiological stress has contributed to heterogeneity of findings in the current review. For example, while studies by Chang et al. [53] and Lyu and Jackson [25] observed increased activity in the insula and amygdala in those with DE, it is possible that heightened activation in these regions is attributable to the ongoing processing of pain from cold water submersion [141], rather than interoceptive or reward-related processing of food-cues.

Furthermore, some studies [66, 67], employed ‘negative affect’ or ‘social evaluation’ stress paradigms. While negative affect and social evaluation are important elements in the acute stress response [142–144], it is possible that such paradigms may not fully capture the elements that contribute to activation of the stress response in naturalistic environments. Therefore, further work in this area may benefit from the strict use of acute ‘stress’ paradigms that can adequately capture the factors that contribute to stress in DE populations (i.e., social evaluation of eating, interpersonal distress, body image concerns) [145–147].

Finally, there was some heterogeneity in fMRI tasks used as dependent measures. While most studies used food-cue reactivity tasks, Jarcho et al. [66] used a social evaluation task, while Hartogsvled et al. [65] used a task that probes goal-directed and habitual responding. While significant differences in the later study were mostly localised to mesocorticolimbic regions, significant differences in activation between DE and control groups in the former were localised in the prefrontal cortex, which plays a role in emotional inhibition and social cognition [148, 149]. This contrasts with the findings of the remaining studies, where most significant activation was localised to regions involved in reward valence and reward-outcome processing. Given interpersonal distress is a trigger for DE episodes [150, 151], it may be worth further exploring similar paradigms to better understand whether impairments in social processing could be an alternative mechanism underpinning stress-related DE.

#### Hormonal and metabolic assessment

Four studies included in the current review measured cortisol. Although cortisol reactivity is not always a reliable indicator of the acute stress response [152], it is generally indicative of levels of cumulative stress [153], and is therefore an important quantitative measure to consider when undertaking an assessment of chronic stress exposure. Although the only study to assess cumulative stress, Tryon et al. [68], did account for diurnal changes in cortisol secretion across both experimental groups, this data was not correlated with any neuroimaging data. This may present a missed opportunity, as evidence suggests that cortisol responsivity influences neural activity [154, 155].

Moreover, regardless of acute or chronic stress exposure, including cortisol reactivity as a covariate in statistical analyses may also be a useful method of understanding interindividual differences in stress-related neural activity in populations characterised by DE, and may also be important in accounting for differences in *ad libitum* food consumption, such as those observed across studies in the current review. Finally, assessing other appetitive hormonal factors, such as ghrelin and leptin, and metabolic comorbidities may also be useful in developing a more comprehensive understanding of the interactions between neural and hormonal regulation of feeding mechanisms in those with DE [156].

#### Integration of acute and chronic stress exposure / measurement

In the context of DE, the influences of acute and chronic stress are often investigated in isolation. This was largely demonstrated in the current review, with only one study considering both the influence of acute and chronic stress on DE. While parsing the effects of acute and chronic stress may aid the practicality of data collection and simplify fMRI interpretation, it fails to account for the complex environment in which eating patterns occur. Generally, those with emotional eating and BED symptoms often experience chronic levels of stress, with acute stress exposure often responsible for the precipitation of unrestrained eating episodes [157]. Therefore, our findings for acute stress cannot account for length of chronic stress exposure, and whether length of exposure may impact relevant neural processes in response to acute stress. Moving forward, future studies may look to adapt comprehensive designs assessing both acute and chronic levels of stress in the interpretation of functional neuroimaging findings.

## Conclusion

Overall, the emerging literature investigating the neural underpinnings of stress and DE present several noteworthy, albeit mixed findings, especially those related to the acute effects of stress. The conclusions drawn from these existing studies are, however, limited by significant methodological heterogeneity and limited sample size. Despite the limited conclusions drawn from this work, by addressing the limitations of existing work, future research can be more cohesive and further demystify this emerging body of literature.

## Electronic supplementary material

Below is the link to the electronic supplementary material.

Supplementary Material 1

## References

[1] Vainik U, García-García I, Dagher A (2019). Uncontrolled eating: a unifying heritable trait linked with obesity, overeating, personality and the brain. Eur J Neurosci.

[2] Stunkard AJ, Messick S (1985). The three-factor eating questionnaire to measure dietary restraint, disinhibition and hunger. J Psychosom Res.

[3] Hays NP, Roberts SB (2008). Aspects of eating Behaviors “Disinhibition” and “Restraint” are related to Weight Gain and BMI in women. Obesity.

[4] Lazarevich I, Irigoyen Camacho ME, Velázquez-Alva MdC, Zepeda Zepeda M. Relationship among obesity, depression, and emotional eating in young adults. Appetite. 2016;107. 10.1016/j.appet.2016.09.011. https://doi.org/https://doi.org/. :639 – 44. 10.1016/j.appet.2016.09.011 27620648

[5] Kessler RC, Berglund PA, Chiu WT, Deitz AC, Hudson JI, Shahly V, the World Health Organization World Mental Health Surveys (2013). The prevalence and correlates of binge eating disorder in. Biol Psychiatry.

[6] de Zwaan M (2001). Binge eating disorder and obesity. Int J Obes.

[7] Woldeyohannes HO, Soczynska JK, Maruschak NA, Syeda K, Wium-Andersen IK, Lee Y (2016). Binge eating in adults with mood disorders: results from the International Mood Disorders Collaborative Project. Obes Res Clin Pract.

[8] Becker DF, Grilo CM (2015). Comorbidity of mood and substance use disorders in patients with binge-eating disorder: Associations with personality disorder and eating disorder pathology. J Psychosom Res.

[9] Rosenbaum DL, White KS (2015). The relation of anxiety, depression, and stress to binge eating behavior. J Health Psychol.

[10] Hart LM, Granillo MT, Jorm AF, Paxton SJ (2011). Unmet need for treatment in the eating disorders: a systematic review of eating disorder specific treatment seeking among community cases. Clin Psychol Rev.

[11] Meany G, Conceição E, Mitchell JE, Binge Eating (2014). Binge eating disorder and loss of Control Eating: Effects on Weight Outcomes after bariatric surgery. Eur Eat Disorders Rev.

[12] Klatzkin RR, Gaffney S, Cyrus K, Bigus E, Brownley KA (2015). Binge eating disorder and obesity: preliminary evidence for distinct cardiovascular and psychological phenotypes. Physiol Behav.

[13] Lazarus RS, Folkman S. Stress, appraisal, and coping. Springer Pub. Co.: New York;; 1984.

[14] Cohen S, Gianaros PJ, Manuck SB (2016). A stage model of stress and disease. Perspect Psychol Sci.

[15] Juster R-P, McEwen BS, Lupien SJ (2010). Allostatic load biomarkers of chronic stress and impact on health and cognition. Neurosci Biobehavioral Reviews.

[16] Suvarna B, Suvarna A, Phillips R, Juster R-P, McDermott B, Sarnyai Z (2020). Health risk behaviours and allostatic load: a systematic review. Neurosci Biobehavioral Reviews.

[17] Guidi J, Lucente M, Sonino N, Fava GA (2021). Allostatic load and its impact on Health: a systematic review. Psychother Psychosom.

[18] Dhabhar FS (2014). Effects of stress on immune function: the good, the bad, and the beautiful. Immunol Res.

[19] Striegel-Moore RH, Dohm F-A, Kraemer HC, Schreiber GB, Taylor CB, Daniels SR (2007). Risk factors for binge-eating disorders: an exploratory study. Int J Eat Disord.

[20] Thurston IB, Hardin R, Kamody RC, Herbozo S, Kaufman C. The moderating role of resilience on the relationship between perceived stress and binge eating symptoms among young adult women. Eat Behav. 2018;29. 10.1016/j.eatbeh.2018.03.009. :114 – 19. 10.1016/j.eatbeh.2018.03.009 29653301

[21] Richardson AS, Arsenault JE, Cates SC, Muth MK (2015). Perceived stress, unhealthy eating behaviors, and severe obesity in low-income women. Nutr J.

[22] Sulkowski ML, Dempsey J, Dempsey AG (2011). Effects of stress and coping on binge eating in female college students. Eat Behav.

[23] Freeman LMY, Gil KM (2004). Daily stress, coping, and dietary restraint in binge eating. Int J Eat Disord.

[24] Spoor STP, Bekker MHJ, Van Strien T, van Heck GL (2007). Relations between negative affect, coping, and emotional eating. Appetite.

[25] Nguyen-Rodriguez ST, Unger JB, Spruijt-Metz D (2009). Psychological determinants of emotional eating in adolescence. Eat Disord.

[26] Gluck ME, Geliebter A, Hung J, Yahav E (2004). Cortisol, hunger, and desire to binge eat following a cold stress test in obese women with binge eating disorder. Psychosom Med.

[27] Gluck ME, Geliebter A, Lorence M (2004). Cortisol stress response is positively correlated with central obesity in obese women with binge eating disorder (BED) before and after cognitive-behavioral treatment. Ann N Y Acad Sci.

[28] Lyu ZY, Jackson T (2016). Acute stressors reduce neural inhibition to food cues and increase eating among binge eating disorder symptomatic women. Front Behav Neurosci.

[29] Klatzkin RR, Gaffney S, Cyrus K, Bigus E, Brownley KA (2018). Stress-induced eating in women with binge-eating disorder and obesity. Biol Psychol.

[30] Schulz S, Laessle RG (2012). Stress-induced laboratory eating behavior in obese women with binge eating disorder. Appetite.

[31] Smith KE, Mason TB, Schaefer LM, Anderson LM, Critchley K, Crosby RD (2021). Dynamic stress responses and real-time symptoms in binge-eating disorder. Ann Behav Med.

[32] Kuijer RG, Boyce JA (2012). Emotional eating and its effect on eating behaviour after a natural disaster. Appetite.

[33] Rutters F, Nieuwenhuizen AG, Lemmens SGT, Born JM, Westerterp-Plantenga MS (2009). Acute stress-related changes in eating in the absence of Hunger. Obesity.

[34] Grilo CM, White MA (2011). A controlled evaluation of the distress criterion for binge eating disorder. J Consult Clin Psychol.

[35] Polivy J, Herman CP (1993). Etiology of binge eating: psychological mechanisms. Binge eating: Nature, assessment, and treatment.

[36] Dingemans A, Danner U, Parks M. Emotion regulation in binge eating disorder: a review. Nutrients. 2017;9(11). 10.3390/nu9111274. 10.3390/nu9111274 PMC5707746 29165348

[37] Leehr EJ, Krohmer K, Schag K, Dresler T, Zipfel S, Giel KE. Emotion regulation model in binge eating disorder and obesity - a systematic review. Neurosci Biobehavioral Reviews. 2015;49. 10.1016/j.neubiorev.2014.12.008. :125 – 34. 10.1016/j.neubiorev.2014.12.008 25530255

[38] Combs JL, Smith GT, Flory K, Simmons JR, Hill KK (2010). The acquired preparedness model of risk for bulimic symptom development. Psychol Addict Behav.

[39] Schell SE, Brassard SL, Racine SE. Extending the Acquired preparedness model of binge eating: testing the indirect effects of high-risk personality traits on binge eating via positive and negative reinforcement expectancies. Appetite. 2019;140. 10.1016/j.appet.2019.05.020. :206 – 12. 10.1016/j.appet.2019.05.020 31102671

[40] Yau YHC, Potenza MN (2013). Stress and eating behaviors. Minerva Endocrinol.

[41] Rutters F, La Fleur S, Lemmens S, Born J, Martens M, Adam T (2012). The hypothalamic-pituitary-adrenal Axis, obesity, and chronic stress exposure: Foods and HPA Axis. Curr Obes Rep.

[42] Herman JP, McKlveen JM, Ghosal S, Kopp B, Wulsin A, Makinson R (2016). Regulation of the hypothalamic-pituitary-adrenocortical stress response. Compr Physiol.

[43] Rouach V, Bloch M, Rosenberg N, Gilad S, Limor R, Stern N (2007). The acute ghrelin response to a psychological stress challenge does not predict the post-stress urge to eat. Psychoneuroendocrinology.

[44] van Strien T, Roelofs K, de Weerth C (2013). Cortisol reactivity and distress-induced emotional eating. Psychoneuroendocrinology.

[45] Rich EL, Romero LM (2005). Exposure to chronic stress downregulates corticosterone responses to acute stressors. Am J Physiology-Regulatory Integr Comp Physiol.

[46] Raspopow K, Abizaid A, Matheson K, Anisman H (2014). Anticipation of a psychosocial stressor differentially influences ghrelin, cortisol and food intake among emotional and non-emotional eaters. Appetite.

[47] Rosenberg N, Bloch M, Ben Avi I, Rouach V, Schreiber S, Stern N (2013). Cortisol response and desire to binge following psychological stress: comparison between obese subjects with and without binge eating disorder. Psychiatry Res.

[48] Schulz S, Laessle R, Hellhammer D (2011). No evidence of increased cortisol stress response in obese women with binge eating disorder. Eating and Weight Disorders - Studies on Anorexia. Bulimia and Obesity.

[49] Kessler RM, Hutson PH, Herman BK, Potenza MN. The neurobiological basis of binge-eating disorder. Neurosci Biobehavioral Reviews. 2016;63. 10.1016/j.neubiorev.2016.01.013. https://doi.org/https://doi.org/. :223 – 38. 10.1016/j.neubiorev.2016.01.013 26850211

[50] Steward T, Menchon JM, Jiménez-Murcia S, Soriano-Mas C, Fernandez-Aranda F (2018). Neural network alterations across eating Disorders: a narrative review of fMRI studies. Curr Neuropharmacol.

[51] Wonderlich JA, Bershad M, Steinglass JE (2021). Exploring neural mechanisms related to cognitive control, reward, and affect in eating Disorders: a narrative review of FMRI Studies. Neuropsychiatr Dis Treat.

[52] Steward T, Miranda-Olivos R, Soriano-Mas C, Fernández-Aranda F (2019). Neuroendocrinological mechanisms underlying impulsive and compulsive behaviors in obesity: a narrative review of fMRI studies. Reviews in Endocrine and Metabolic Disorders.

[53] Balodis IM, Grilo CM, Potenza MN (2015). Neurobiological features of binge eating disorder. CNS Spectr.

[54] Balodis IM, Kober H, Worhunsky PD, White MA, Stevens MC, Pearlson GD (2013). Monetary reward Processing in obese individuals with and without binge eating disorder. Biol Psychiatry.

[55] Nakamura Y, Koike S (2021). Association of Disinhibited Eating and Trait of Impulsivity with Insula and Amygdala responses to palatable liquid consumption. Front Syst Neurosci.

[56] Steward T, Pico-Perez M, Mata F, Martinez-Zalacain I, Cano M, Contreras-Rodriguez O (2016). Emotion regulation and excess weight: impaired affective processing characterized by dysfunctional insula activation and connectivity. PLoS ONE.

[57] Steward T, Picó-Pérez M, Mestre-Bach G, Martínez-Zalacaín I, Suñol M, Jiménez-Murcia S (2019). A multimodal MRI study of the neural mechanisms of emotion regulation impairment in women with obesity. Translational Psychiatry.

[58] Wood SMW, Schembre SM, He Q, Engelmann JM, Ames SL, Bechara A. Emotional eating and routine restraint scores are associated with activity in brain regions involved in urge and self-control. Physiol Behav. 2016;165. 10.1016/j.physbeh.2016.08.024. :405 – 12. 10.1016/j.physbeh.2016.08.024 PMC5036966 27575974

[59] Berretz G, Packheiser J, Kumsta R, Wolf OT, Ocklenburg S (2021). The brain under stress—A systematic review and activation likelihood estimation meta-analysis of changes in BOLD signal associated with acute stress exposure. Neurosci Biobehavioral Reviews.

[60] Liston C, McEwen BS, Casey B (2009). Psychosocial stress reversibly disrupts prefrontal processing and attentional control. Proc Natl Acad Sci.

[61] Ironside M, Kumar P, Kang M-S, Pizzagalli DA (2018). Brain mechanisms mediating effects of stress on reward sensitivity. Curr Opin Behav Sci.

[62] Pizzagalli DA (2014). Depression, stress, and anhedonia: toward a synthesis and integrated model. Annu Rev Clin Psychol.

[63] Carnell S, Benson L, Papantoni A, Chen L, Huo Y, Wang Z (2022). Obesity and acute stress modulate appetite and neural responses in food word reactivity task. PLoS ONE.

[64] Chang RS, Cerit H, Hye T, Durham EL, Aizley H, Boukezzi S (2022). Stress-induced alterations in HPA-axis reactivity and mesolimbic reward activation in individuals with emotional eating. Appetite.

[65] Hartogsveld B, Quaedflieg CWEM, van Ruitenbeek P, Smeets T (2022). Decreased putamen activation in balancing goal-directed and habitual behavior in binge eating disorder. Psychoneuroendocrinology.

[66] Jarcho JM, Tanofsky-Kraff M, Nelson EE, Engel SG, Vannucci A, Field SE (2015). Neural activation during anticipated peer evaluation and laboratory meal intake in overweight girls with and without loss of control eating. NeuroImage.

[67] Wagner DD, Boswell RG, Kelley WM, Heatherton TF (2012). Inducing negative affect increases the reward value of Appetizing Foods in Dieters. J Cogn Neurosci.

[68] Tryon MS, Carter CS, DeCant R, Laugero KD (2013). Chronic stress exposure may affect the brain’s response to high calorie food cues and predispose to obesogenic eating habits. Physiol Behav.

[69] Wheaton B. A checklist of ongoing difficult situations in domains of work, relationships, and financial strain. Stress and mental health: Contemporary issues and prospects for the future. 1994:77–114.

[70] Hines EA, Brown GE (1936). The cold pressor test for measuring the reactibility of the blood pressure: data concerning 571 normal and hypertensive subjects. Am Heart J.

[71] Smeets T, Cornelisse S, Quaedflieg CWEM, Meyer T, Jelicic M, Merckelbach H (2012). Introducing the Maastricht Acute stress test (MAST): a quick and non-invasive approach to elicit robust autonomic and glucocorticoid stress responses. Psychoneuroendocrinology.

[72] Velten E (1968). A laboratory task for induction of mood states. Behav Res Ther.

[73] Guyer AE, McClure-Tone EB, Shiffrin ND, Pine DS, Nelson EE (2009). Probing the neural Correlates of anticipated peer evaluation in adolescence. Child Dev.

[74] Guyer AE, Choate VR, Pine DS, Nelson EE (2012). Neural circuitry underlying affective response to peer feedback in adolescence. Soc Cogn Affect Neurosci.

[75] Kirschbaum C, Pirke KM, Hellhammer DH (1993). The ‘Trier Social stress test’ – a Tool for investigating psychobiological stress responses in a laboratory setting. Neuropsychobiology.

[76] Chang RS, Cerit H, Hye T, Durham EL, Aizley H, Boukezzi S (2022). Stress-induced alterations in HPA-axis reactivity and mesolimbic reward activation in individuals with emotional eating. Appetite.

[77] Collins B, Breithaupt L, McDowell JE, Miller LS, Thompson J, Fischer S (2017). The impact of Acute stress on the neural Processing of Food Cues in Bulimia Nervosa: replication in two samples. J Abnorm Psychol.

[78] Fischer S, Breithaupt L, Wonderlich J, Westwater ML, Crosby RD, Engel SG (2017). Impact of the neural correlates of stress and cue reactivity on stress related binge eating in the natural environment. J Psychiatr Res.

[79] Westwater ML, Mancini F, Gorka AX, Shapleske J, Serfontein J, Grillon C (2021). Prefrontal responses during proactive and reactive inhibition are differentially impacted by stress in Anorexia and Bulimia Nervosa. J Neurosci.

[80] Dreyfuss MFW, Riegel ML, Pedersen GA, Cohen AO, Silverman MR, Dyke JP (2017). Patients with bulimia nervosa do not show typical neurodevelopment of cognitive control under emotional influences. Psychiatry Research-Neuroimaging.

[81] Wonderlich JA, Breithaupt L, Thompson JC, Crosby RD, Engel SG, Fischer S (2018). The impact of neural responses to food cues following stress on trajectories of negative and positive affect and binge eating in daily life. J Psychiatr Res.

[82] Leenaerts N, Jongen D, Ceccarini J, Van Oudenhove L, Vrieze E (2022). The neurobiological reward system and binge eating: a critical systematic review of neuroimaging studies. Int J Eat Disord.

[83] Meye FJ, Adan RAH (2014). Feelings about food: the ventral tegmental area in food reward and emotional eating. Trends Pharmacol Sci.

[84] Bohon C, Stice E, Spoor S (2009). Female emotional eaters show abnormalities in consummatory and anticipatory food reward: a functional magnetic resonance imaging study. Int J Eat Disord.

[85] Goldschmidt AB, Dickstein DP, MacNamara AE, Phan KL, O’Brien S, Le Grange D (2018). A pilot study of neural Correlates of loss of Control Eating in Children with Overweight/Obesity: probing Intermittent Access to Food as a Means of eliciting disinhibited eating. J Pediatr Psychol.

[86] Kumar P, Berghorst LH, Nickerson LD, Dutra SJ, Goer FK, Greve DN (2014). Differential effects of acute stress on anticipatory and consummatory phases of reward processing. Neuroscience.

[87] Balodis IM, Grilo CM, Kober H, Worhunsky PD, White MA, Stevens MC (2014). A pilot study linking reduced fronto–striatal recruitment during reward processing to persistent bingeing following treatment for binge-eating disorder. Int J Eat Disord.

[88] Simon JJ, Skunde M, Walther S, Bendszus M, Herzog W, Friederich H-C (2016). Neural signature of food reward processing in bulimic-type eating disorders. Soc Cogn Affect Neurosci.

[89] Kung P-H, Soriano-Mas C, Steward T (2022). The influence of the subcortex and brain stem on overeating: how advances in functional neuroimaging can be applied to expand neurobiological models to beyond the cortex. Reviews in Endocrine and Metabolic Disorders.

[90] Born JM, Lemmens SGT, Rutters F, Nieuwenhuizen AG, Formisano E, Goebel R (2010). Acute stress and food-related reward activation in the brain during food choice during eating in the absence of hunger. Int J Obes.

[91] Henze G-I, Zänkert S, Urschler DF, Hiltl TJ, Kudielka BM, Pruessner JC (2017). Testing the ecological validity of the Trier social stress test: Association with real-life exam stress. Psychoneuroendocrinology.

[92] Quaedflieg CWEM, Meyer T, van Ruitenbeek P, Smeets T. Examining habituation and sensitization across repetitive laboratory stress inductions using the MAST. Psychoneuroendocrinology. 2017;77. 10.1016/j.psyneuen.2016.12.009. https://doi.org/https://doi.org/. :175 – 81. 10.1016/j.psyneuen.2016.12.009 28068575

[93] Gadea M, Gómez C, González-Bono E, Espert R, Salvador A (2005). Increased cortisol and decreased right ear advantage (REA) in dichotic listening following a negative mood induction. Psychoneuroendocrinology.

[94] Salas C, Radovic D, Turnbull O, Inside-Out. Comparing internally generated and externally generated Basic Emotions. Emot (Washington DC). 2011;12. 10.1037/a0025811. :568 – 78. 10.1037/a0025811 22023364

[95] Klatzkin RR, Dasani R, Warren M, Cattaneo C, Nadel T, Nikodem C (2019). Negative affect is associated with increased stress-eating for women with high perceived life stress. Physiol Behav.

[96] Bohon C, Stice E (2012). Negative affect and neural response to palatable food intake in bulimia nervosa. Appetite.

[97] Hampshire A, Chamberlain SR, Monti MM, Duncan J, Owen AM (2010). The role of the right inferior frontal gyrus: inhibition and attentional control. NeuroImage.

[98] Rae CL, Hughes LE, Anderson MC, Rowe JB (2015). The Prefrontal Cortex achieves Inhibitory Control by facilitating Subcortical Motor Pathway Connectivity. J Neurosci.

[99] Weidong C, Srikanth R, Tianwen C, Chiang-Shan RL, Vinod M (2014). Dissociable roles of right Inferior Frontal Cortex and Anterior Insula in Inhibitory Control: evidence from intrinsic and Task-Related Functional Parcellation, Connectivity, and Response Profile analyses across multiple datasets. J Neurosci.

[100] Zald DH, Mattson DL, Pardo JV. Brain activity in ventromedial prefrontal cortex correlates with individual differences in negative affect. Proceedings of the National Academy of Sciences. 2002;99(4):2450-54; 10.1073/pnas.042457199. 10.1073/pnas.042457199 PMC122385 11842195

[101] Myers-Schulz B, Koenigs M (2012). Functional anatomy of ventromedial prefrontal cortex: implications for mood and anxiety disorders. Mol Psychiatry.

[102] Silvers JA, Insel C, Powers A, Franz P, Helion C, Martin RE (2017). vlPFC–vmPFC–Amygdala interactions underlie age-related differences in cognitive regulation of emotion. Cereb Cortex.

[103] Zhao J, Mo L, Bi R, He Z, Chen Y, Xu F (2021). The VLPFC versus the DLPFC in Downregulating Social Pain using reappraisal and distraction strategies. J Neurosci.

[104] Cristofori I, Moretti L, Harquel S, Posada A, Deiana G, Isnard J (2013). Theta Signal as the neural signature of Social Exclusion. Cereb Cortex.

[105] van Marle HJF, Hermans EJ, Qin S, Fernández G (2010). Enhanced resting-state connectivity of amygdala in the immediate aftermath of acute psychological stress. NeuroImage.

[106] Kondo H, Osaka N, Osaka M (2004). Cooperation of the anterior cingulate cortex and dorsolateral prefrontal cortex for attention shifting. NeuroImage.

[107] Quigley KS, Kanoski S, Grill WM, Barrett LF, Tsakiris M (2021). Functions of Interoception: from Energy Regulation to experience of the self. Trends Neurosci.

[108] Kim C, Kim S, Park S (2017). Neurogenic effects of ghrelin on the hippocampus. Int J Mol Sci.

[109] Beck B, Pourié G (2013). Ghrelin, neuropeptide Y, and other feeding-regulatory peptides active in the hippocampus: role in learning and memory. Nutr Rev.

[110] Hsu TM, Hahn JD, Konanur VR, Noble EE, Suarez AN, Thai J (2015). Hippocampus ghrelin signaling mediates appetite through lateral hypothalamic orexin pathways. Elife.

[111] Jenkinson PM, Taylor L, Laws KR (2018). Self-reported interoceptive deficits in eating disorders: a meta-analysis of studies using the eating disorder inventory. J Psychosom Res.

[112] Young HA, Williams C, Pink AE, Freegard G, Owens A, Benton D (2017). Getting to the heart of the matter: does aberrant interoceptive processing contribute towards emotional eating?. PLoS ONE.

[113] Ahlich E, Rancourt D (2022). Boredom proneness, interoception, and emotional eating. Appetite.

[114] Petzschner FH, Garfinkel SN, Paulus MP, Koch C, Khalsa SS (2021). Computational models of Interoception and Body Regulation. Trends Neurosci.

[115] Yau YH, Potenza MN (2013). Stress and eating behaviors. Minerva Endocrinol.

[116] Adam TC, Epel ES (2007). Stress, eating and the reward system. Physiol Behav.

[117] Lupien SJ, Juster R-P, Raymond C, Marin M-F (2018). The effects of chronic stress on the human brain: from neurotoxicity, to vulnerability, to opportunity. Front Neuroendocrinol.

[118] Schag K, Schönleber J, Teufel M, Zipfel S, Giel KE (2013). Food-related impulsivity in obesity and binge eating disorder – a systematic review. Obes Rev.

[119] Macht M (2008). How emotions affect eating: a five-way model. Appetite.

[120] Sinha R, Jastreboff AM (2013). Stress as a common risk factor for obesity and addiction. Biol Psychiatry.

[121] Ester T, Kullmann S (2022). Neurobiological regulation of eating behavior: evidence based on non-invasive brain stimulation. Reviews in Endocrine and Metabolic Disorders.

[122] Suzuki S, Cross L, O’Doherty JP (2017). Elucidating the underlying components of food valuation in the human orbitofrontal cortex. Nat Neurosci.

[123] Ye BS, Jeon S, Yoon S, Kang SW, Baik K, Lee Y (2018). Effects of dopaminergic depletion and brain atrophy on neuropsychiatric symptoms in de novo Parkinson’s disease. J Neurol Neurosurg Psychiatry.

[124] Möschl M, Schmidt K, Enge S, Weckesser LJ, Miller R (2022). Chronic stress and executive functioning: a specification-curve analysis. Physiol Behav.

[125] Friedman A, Homma D, Bloem B, Gibb LG, Amemori K-i, Hu D (2017). Chronic stress alters striosome-circuit dynamics, leading to aberrant decision-making. Cell.

[126] Ottino-González J, Jurado MA, García-García I, Caldú X, Prats-Soteras X, Tor E (2019). Allostatic load and executive functions in overweight adults. Psychoneuroendocrinology.

[127] Rouel M, Raman J, Hay P, Smith E (2016). Validation of the Behaviour rating inventory of executive function–adult version (BRIEF-A) in the obese with and without binge eating disorder. Eat Behav.

[128] Navas JF, Vilar-López R, Perales JC, Steward T, Fernández-Aranda F, Verdejo-García A (2016). Altered decision-making under risk in obesity. PLoS ONE.

[129] Miranda-Olivos R, Steward T, Martínez-Zalacaín I, Mestre-Bach G, Juaneda-Seguí A, Jiménez-Murcia S (2021). The neural correlates of delay discounting in obesity and binge eating disorder. J Behav Addictions.

[130] Ramnani N, Owen AM (2004). Anterior prefrontal cortex: insights into function from anatomy and neuroimaging. Nat Rev Neurosci.

[131] Volman I, Roelofs K, Koch S, Verhagen L, Toni I (2011). Anterior prefrontal cortex inhibition impairs control over social emotional actions. Curr Biol.

[132] Smith DG, Robbins TW (2013). The neurobiological underpinnings of obesity and binge eating: a rationale for adopting the Food Addiction Model. Biol Psychiatry.

[133] Turton R, Chami R, Treasure J, Emotional, Eating, Binge Eating and Animal Models of Binge-Type Eating Disorders (2017). Curr Obes Rep.

[134] Tan CC, Chow CM (2014). Stress and emotional eating: the mediating role of eating dysregulation. Pers Indiv Differ.

[135] Groesz LM, McCoy S, Carl J, Saslow L, Stewart J, Adler N (2012). What is eating you? Stress and the drive to eat. Appetite.

[136] O’Neill J, Kamper-DeMarco K, Chen X, Orom H (2020). Too stressed to self-regulate? Associations between stress, self-reported executive function, disinhibited eating, and BMI in women. Eat Behav.

[137] Razzoli M, Bartolomucci A (2016). The dichotomous effect of chronic stress on obesity. Trends in Endocrinology & Metabolism.

[138] Tomiyama AJ, Stress, Obesity (2019). Ann Rev Psychol.

[139] Berner LA, Brown TA, Lavender JM, Lopez E, Wierenga CE, Kaye WH (2019). Neuroendocrinology of reward in anorexia nervosa and bulimia nervosa: beyond leptin and ghrelin. Mol Cell Endocrinol.

[140] Vainik U, Neseliler S, Konstabel K, Fellows LK, Dagher A. Eating traits questionnaires as a continuum of a single concept. Uncontrolled eating. Appetite. 2015;90. 10.1016/j.appet.2015.03.004. :229 – 39. 10.1016/j.appet.2015.03.004 25769975

[141] Kogler L, Müller VI, Chang A, Eickhoff SB, Fox PT, Gur RC (2015). Psychosocial versus physiological stress - Meta-analyses on deactivations and activations of the neural correlates of stress reactions. NeuroImage.

[142] Feldman PJ, Cohen S, Lepore SJ, Matthews KA, Kamarck TW, Marsland AL (1999). Negative emotions and acute physiological responses to stress. Ann Behav Med.

[143] Dickerson SS, Gruenewald TL, Kemeny ME (2009). Psychobiological responses to Social Self threat: functional or detrimental?. Self and Identity.

[144] Dickerson SS, Mycek PJ, Zaldivar F (2008). Negative social evaluation, but not mere social presence, elicits cortisol responses to a laboratory stressor task. Health Psychol.

[145] Ansell EB, Grilo CM, White MA (2012). Examining the interpersonal model of binge eating and loss of control over eating in women. Int J Eat Disord.

[146] O’Connor DB, Jones F, Conner M, McMillan B, Ferguson E (2008). Effects of daily hassles and eating style on eating behavior. Health Psychol.

[147] Sawaoka T, Barnes RD, Blomquist KK, Masheb RM, Grilo CM (2012). Social anxiety and self-consciousness in binge eating disorder: associations with eating disorder psychopathology. Compr Psychiatr.

[148] Forbes CE, Grafman J (2010). The role of the human prefrontal cortex in Social Cognition and Moral Judgment. Annu Rev Neurosci.

[149] Weissman DH, Perkins AS, Woldorff MG (2008). Cognitive control in social situations: a role for the dorsolateral prefrontal cortex. NeuroImage.

[150] Yu J, Selby EA. The interaction between affective lability and interpersonal problems in binge eating. J Soc Clin Psychol. 2013(5):465–81.

[151] Ambwani S, Roche MJ, Minnick AM, Pincus AL (2015). Negative affect, interpersonal perception, and binge eating behavior: an experience sampling study. Int J Eat Disord.

[152] Miller R, Plessow F, Kirschbaum C, Stalder T. Classification criteria for distinguishing Cortisol Responders from Nonresponders to Psychosocial stress: evaluation of salivary cortisol pulse detection in panel designs. Psychosom Med. 2013;75(9), https://journals.lww.com/psychosomaticmedicine/Fulltext/2013/11000/Classification_Criteria_for_Distinguishing.6.aspx. 10.1097/PSY.0000000000000002 24184845

[153] Lee DY, Kim E, Choi MH (2015). Technical and clinical aspects of cortisol as a biochemical marker of chronic stress. BMB Rep.

[154] Dimitrov A, Demin K, Fehlner P, Walter H, Erk S, Veer IM (2018). Differences in neural recovery from acute stress between Cortisol Responders and Non-responders. Front Psychiatry.

[155] Harrewijn A, Vidal-Ribas P, Clore-Gronenborn K, Jackson SM, Pisano S, Pine DS (2020). Associations between brain activity and endogenous and exogenous cortisol – a systematic review. Psychoneuroendocrinology.

[156] García-García I, Michaud A, Jurado M, Dagher A, Morys F (2022). Mechanisms linking obesity and its metabolic comorbidities with cerebral grey and white matter changes. Reviews in Endocrine and Metabolic Disorders.

[157] Naish KR, Laliberte M, MacKillop J, Balodis IM (2019). Systematic review of the effects of acute stress in binge eating disorder. Eur J Neurosci.

