# Supplementary material for: The influence of stress on the neural underpinnings of disinhibited eating: a systematic review and future directions for research
Source: Rev Endocr Metab Disord. 2023 Jun 13;24(4):713–34. doi: 10.1007/s11154-023-09814-4 (PMC10404573; doi:10.1007/s11154-023-09814-4)
Supplement: Supplementary file 1 — Supplementary Material 1 [file 11154_2023_9814_MOESM1_ESM.pdf]

Supplementary Information

Title: The influence of stress on the neural underpinnings of disinhibited eating: A systematic review and future directions for research.

Journal: Reviews in Endocrine and Metabolic Disorders.

Authors: Emily Giddens, Brittany Noy, Trevor Steward, Antonio Verdejo-García.

Corresponding author: Antonio Verdejo-García,

Turner Institute for Brain and Mental Health, Monash University,

Antonio.verdejo@monash.edu

Table of Contents

Figures
Supplementary Figure 1 page 2

Tables
Supplementary table 1 page 3
Supplementary table 2 page 6
Supplementary table 3 page 7

Supplementary figure 1.

PRISMA flow chart of article selection process for the exploratory review.

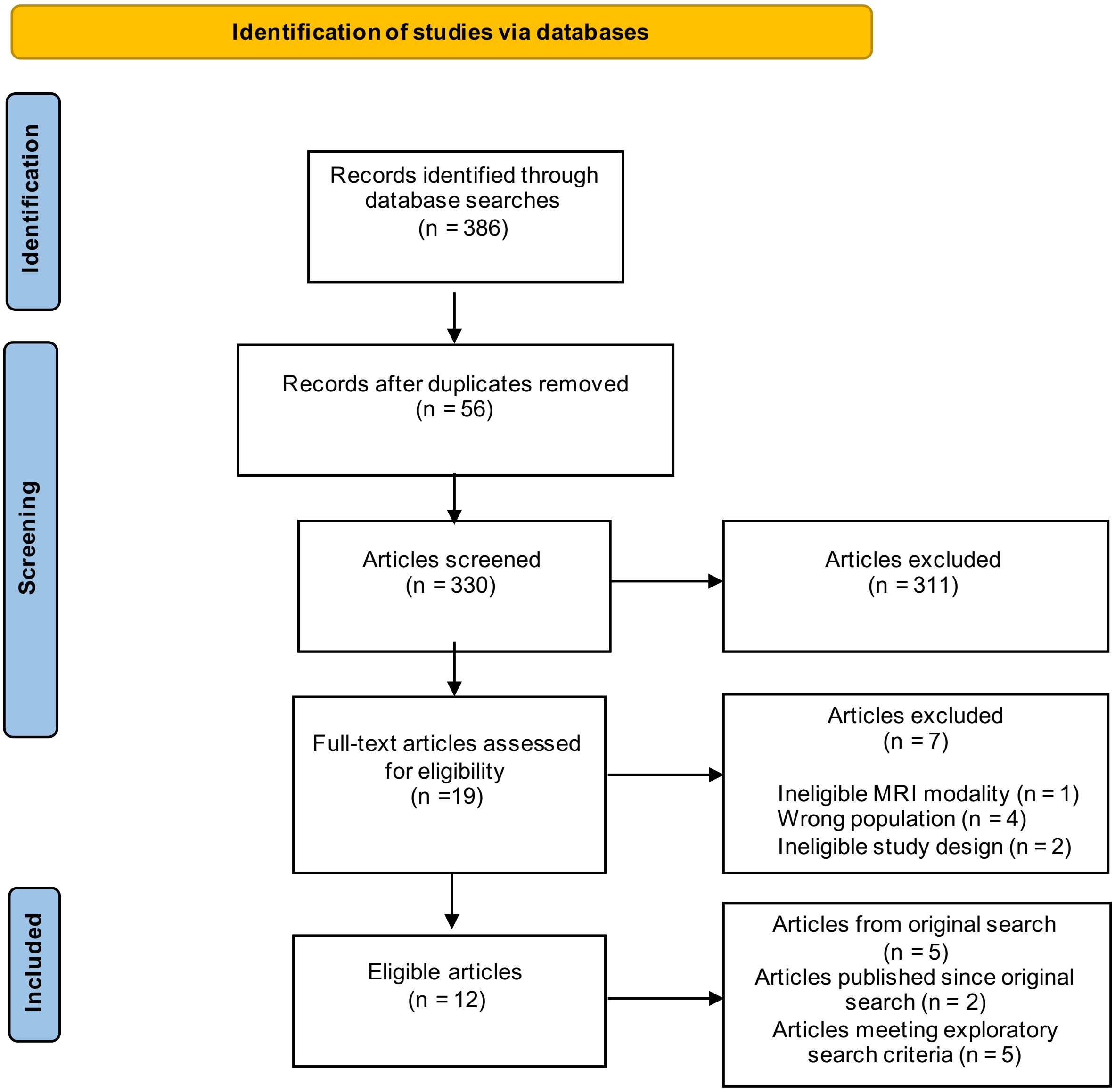

Supplementary table 1.

Comprehensive search strategies and output by database.

Database name	Date searched	#	Search	Results
MEDLINE® Ovid	3/1/22	1	“binge eating”.ti,ab. OR “binge eating disorder”.ti,ab. OR “binge eating scale”.ti,ab. OR “eating disorder examination questionnaire”.ti,ab. OR “subthreshold bing*”.ti,ab. OR “emotional eating”.ti,ab. OR “uncontrol* eating”.ti,ab. OR “loss of control”.ti,ab. OR “LOC”.ti,ab. OR “opportunistic eating”.ti,ab. OR “external eating”.ti,ab. OR “disinhibition”.ti,ab. OR “disinhibited eating”.ti,ab. OR food craving”.ti,ab. OR “hedonic eating”.ti,ab. OR hedonic hunger”.ti,ab. OR “reward-based eating”.ti,ab.	12,843
		2	“acute stress*”.ti,ab. OR “daily stress*”.ti,ab. OR “transient stress*”.ti,ab. OR “cortisol”.ti,ab. OR “stress test”.ti,ab. OR “stress paradigm”.ti,ab. OR “negative affect*”.ti,ab. OR “Trier Social Stress Test”.ti,ab. OR “Cold Pressor Test”.ti,ab. OR “arithmetic task”.ti,ab. OR “interpersonal stress”.ti,ab. OR “social evaluation”.ti,ab. OR “HPA”.ti,ab. OR “hypothalamic pituitary adrenal.ti,ab. OR “chronic stress*”.ti,ab. OR “hair cortisol” or “saliv* cortisol”.ti,ab. OR “cumulative stress*”.ti,ab. OR “allostasis”.ti,ab. OR “allostatic load”.ti,ab.	110,351
		3	“functional neuroimag*” or “fMRI” or “MRI” or “white matter” OR “gr?y matter” or “brain volume” OR “BOLD”	317,519
		4	1 AND 2 AND 3	14
PsychInfo® Ovid	3/1/22	1	“binge eating”.ti,ab. OR “binge eating disorder”.ti,ab. OR “binge eating scale”.ti,ab. OR “eating disorder examination questionnaire”.ti,ab. OR “subthreshold bing*”.ti,ab. OR “emotional eating”.ti,ab. OR “uncontrol* eating”.ti,ab. OR “loss of control”.ti,ab. OR “LOC”.ti,ab. OR “opportunistic eating”.ti,ab. OR “external eating”.ti,ab. OR “disinhibition”.ti,ab. OR “disinhibited eating”.ti,ab. OR food craving”.ti,ab. OR “hedonic eating”.ti,ab. OR hedonic hunger”.ti,ab. OR “reward-based eating”.ti,ab.	12,689
		2	“acute stress*”.ti,ab. OR “daily stress*”.ti,ab. OR “transient stress*”.ti,ab. OR “cortisol”.ti,ab. OR “stress test”.ti,ab. OR “stress paradigm”.ti,ab. OR “negative affect*”.ti,ab. OR “Trier Social Stress Test”.ti,ab. OR “Cold Pressor Test”.ti,ab. OR “arithmetic task”.ti,ab. OR “interpersonal stress”.ti,ab. OR “social evaluation”.ti,ab. OR “HPA”.ti,ab. OR “hypothalamic pituitary adrenal.ti,ab. OR “chronic stress*”.ti,ab. OR “hair cortisol” or “saliv* cortisol”.ti,ab. OR “cumulative stress*”.ti,ab. OR “allostasis”.ti,ab. OR “allostatic load”.ti,ab.	57,805
		3	“functional neuroimag*” or “fMRI” or “MRI” or “white matter” OR “gr?y matter” or “brain volume” OR “BOLD”	84,810
		4	1 AND 2 AND 3	18

Scopus	3/1/22	1	(TITLE-ABS-KEY ("binge eating") OR TITLE-ABS-KEY ("binge eating disorder") OR TITLEABS-KEY ("binge eating scale") OR TITLE-ABSKEY ("eating disorder examination questionnaire") OR TITLE-ABS-KEY ("subthreshold bing*") OR TITLE-ABS-KEY ("emotional eating") OR TITLE-ABS-KEY ("uncontroll* eating") OR TITLE-ABS-KEY ("loss of control") OR TITLE-ABS-KEY ("LOC") OR TITLE-ABS-KEY ("opportunistic eating") OR TITLE-ABS-KEY ("external eating") OR TITLE-ABS-KEY ("disinhibition") OR TITLE-ABS-KEY ("disinhibited eating") OR TITLE-ABSKEY ("food craving") OR TITLE-ABS-KEY ("hedonic eating") OR TITLE-ABS-KEY ("hedonic hunger") OR TITLE-ABS-KEY ("reward-based eating"))	36,767
		2	(TITLE-ABS-KEY ("acute stress*") OR TITLE-ABS-KEY ("daily stress*") OR TITLE-ABS-KEY ("transient stress*") OR TITLE-ABS-KEY ("cortisol") OR TITLE-ABS-KEY ("stress test") OR TITLE-ABS-KEY ("stress paradigm") OR TITLEABS-KEY ("Trier Social Stress Test") OR TITLE-ABS-KEY ("Cold Pressor Test") OR TITLE-ABS-KEY ("Arithmetic task") OR TITLE-ABS-KEY ("negative affect*") OR TITLE-ABS-KEY ("interpersonal stress") OR TITLE-ABS-KEY ("social evaluation") OR TITLE-ABS-KEY ("HPA") OR TITLE-ABS-KEY ("hypothalamic pituitary adrenal") OR TITLE-ABS-KEY (chronic stress*) OR TITLE-ABS-KEY (“hair cortisol”) OR TITLE-ABS-KEY (saliv* cortisol”) OR TITLE-ABS-KEY (“allostatic load”) OR TITLE-ABS-KEY (“allostasis”) OR TITLE-ABS-KEY (“cumulative stress”)	193,020
			(TITLE-ABS-KEY ("functional neuroimag*") OR TITLE-ABSKEY ("fMRI") OR TITLE-ABS-KEY ("MRI") OR TITLE-ABS-KEY ("white matter") OR TITLE-ABS-KEY ("gr?y matter") OR TITLE-ABS-KEY ("BOLD")	529,862
		4	1 AND 2 AND 3	39
Web of Science Core Collection	3/1/22	1	AB=(binge eating) OR AB=(binge eating disorder) OR AB=(Binge eating scale) OR AB=(Eating disorder examination questionnaire) OR AB=(Three Factor Eating Questionnaire) OR AB=(Subthreshold bing*) OR AB=(EDNOS) OR AB=(emotional eating) OR AB=(uncontroll* eating) OR AB=(loss of control) OR AB=(LOC) OR AB=(opportunistic eating) OR AB=(external eating) OR AB=(disinhibition) OR AB=(food craving) OR AB=(hedonic eating) or AB =(hedonic hunger) OR AB=(reward based eating)	337,192
		2	AB=(acute stress*) OR AB=(daily stress*) OR AB=(transient stress*) OR AB=(cortisol) OR AB=(stress test) OR AB=(stress paragidm) OR AB=(negative affect*) OR AB=(Trier Social Stress Test) OR AB=(Cold Pressor Test) OR AB=(arithmetic task)	801,672

			OR AB=(interpersonal stress) OR AB=(social evaluation) OR AB=(hypothalamic pituitary adrenal) OR AB=(HPA) OR AB=(allostatic load) OR AB=(allostasis) OR AB=(chronic stress*) OR AB=(hair cortisol) OR AB=(saliv* cortisol) OR AB=(cumulative stress)	
		3	AB=(fMRI) OR AB=(functional neuroimag*) OR AB=(MRI) OR AB=(white matter) OR AB=(gr?y matter) OR AB=(BOLD)	359,165
		4	1 AND 2 AND 3	176
CINAHL	3/1/22	1	AB binge eating OR AB binge eating disorder OR AB binge eating scale OR AB eating disorder examination questionnaire OR AB subthreshold bing* OR AB emotional eating OR AB uncontroll* eating OR AB loss of control OR AB "LOC" OR AB opportunistic eating OR AB external eating OR AB disinhibition OR AB disinhibited eating OR AB food craving OR AB hedonic eating OR AB hedonic hunger OR AB rewardbased eating	13,417
		2	AB "acute stress*" OR AB "daily stress*" OR AB "transient stress*" OR AB "cortisol" OR AB "stress test" OR AB "stress paradigm" OR AB "Trier Social Stress Test" OR AB "Cold Pressor Test" OR AB "Arithmetic task" OR AB "negative affect*" OR AB "interpersonal stress*" OR AB "social evaluation" OR AB "chronic stress*" OR AB "hair cortisol" OR AB "saliv* cortisol" OR AB "allostatic load" OR AB "allostasis" OR AB “cumulative stress*”	20,220
		3	"functional neuroimag*" OR AB "fMRI” OR AB “MRI” OR AB "white matter" OR AB "gr?y matter" OR AB “BOLD”	69,713
		4	1 AND 2 AND 3	8

Supplementary table 2.

Key search terms by construct used in the exploratory literature search.

Construct	Keywords
Disinhibited eating	"binge eating" OR "binge eating disorder" OR "binge eating scale" OR "eating disorder examination questionnaire" OR "subthreshold bing*" OR "emotional eating" OR "uncontrol* eating" OR "loss of control" OR "LOC" OR "opportunistic eating" OR "external eating" OR "disinhibition" OR "disinhibited eating" OR food craving" OR "hedonic eating" OR hedonic hunger" OR "reward-based eating", OR "bulimia nervosa", OR "binge-purge", OR "night eating syndrome".
Stress	"acute stress*" OR "daily stress*" OR "transient stress*" OR "cortisol" OR "stress test" OR "stress paradigm" OR "negative affect*" OR "Trier Social Stress Test" OR<br><br>"Cold Pressor Test" OR "arithmetic task" OR "interpersonal stress" OR "social evaluation" OR "HPA" OR "hypothalamic pituitary adrenal" OR "chronic stress*" OR "hair cortisol" or "saliv* cortisol" OR "cumulative stress*" OR "allostasis" OR "allostatic load".
Functional neuroimaging	functional neuroimag*" or "fMRI" or "MRI" or "white matter" OR "gr?y matter" or "brain volume" or "BOLD".

Supplementary table 3.

Exploratory search: Summary of study characteristics and main findings.

**Reference**	**Country**	**Sample**	**Disinhibited eating measure**	**Stressor**	**Functional task**	**Stress measurement**	**Variables controlled for**	**Main behavioural findings**	**Main fMRI findings**
Collins et al. (2017) [1].	United States.	*Study 1*: 10 adults with BN (Age: *M* = 21, *SD* = 2.5; BMI: *M* = 21.75, *SD* = 1.59), and 10 healthy controls (Age: *M* = 24.0, *SD* = 5.5; BMI: *M* = 22.21, *SD* = 1.28).<br><br>*Study 2:* 17 BN-symptomatic adults (Age: *M* = 22.85, *SD* = 5.42; BMI: *M* = 24.47, *SD* = 3.25).	*Studies 1 & 2:* SCID-I, EDE.	*Studies 1 & 2:* publicly evaluated serial subtraction task.	*Studies 1 & 2:* observation of high-calorie, low-calorie and control (neutral) stimuli.	*Studies 1 & 2:* self-report (VAS).	*Studies 1 & 2:* BMI, sex, hunger.	*NA.*	*Study 1:* Acute stress in the BN group was associated with increased activation in the occipital lobes and cuneus, and decreased activation in the anterior vermis of the cerebellum and paracingulate gyrus when viewing high-calorie food-cues, relative to healthy controls.<br><br>*Study 2*: BN-symptomatic participants exhibited decreased activation in the right anterior vermis of the cerebellum, paracingulate gyrus and left precuneus when viewing high-calorie relative to low calorie foods during acute stress.

Dreyfuss et al. (2018) [2].	United States.	22 adults with BN (Age: *M* = 25.11, *SD* = 3.98) and 30 healthy controls (Age: *M* = 23.13, *SD* = 3.98).	Clinical interview for BN symptoms, SCID-1, EDE.	CCUE (emotional go / no-go) task.	*As previous*	Self-report (VAS).	Age, sex, duration of BN, frequency of BE episodes, medication.	Participants with BN made more correct responses under positive affect relative to controls. No significant differences in response accuracy were observed between groups during the negative affect state. Neither BN nor healthy control participants demonstrated significant differences in response accuracy to neutral or emotional cues under either affective condition.<br><br>Increased age was associated with greater accuracy on both go and no-go trials in healthy controls, however no improvement with age was observed in BN participants.	Older age predicted greater recruitment of the subgenual cingulate cortex and left MFG during successful task performance in healthy controls.<br><br>Older age predicted diminished recruitment of these MFG and subgenual gyrus during successful task performance in the BN group.
Fischer et al. (2017) [3].	United States.	16 BN-symptomatic adults (Age: *M* = 22.85, *SD* = 5.42; BMI: *M* = 24.27, *SD* = 3.25).	SCID-I, EDE, EMA.	Adapted TSST (administered in scanner).	Observation of highly palatable food-cues and control (neutral) stimuli.	Self-report (VAS, EMA).	BMI, age, hunger.	EMA-reported stress levels increased significantly prior to, and significantly decreased following, self-reported binge-eating episodes.	Acute stress induction was associated with significant decreases in bilateral vmPFC, right ACC, left amygdala in response to high-calorie food cues.<br><br>Participants with significant increases in EMA-reported stress prior

									to binge-eating episodes demonstrated decreased activation of the bilateral precuneus, ACC, and dlPFC in response to palatable foods, relative to those with non-significant changes in stress prior to binging.
Westwater et al. (2021) [4].	United States.	22 adults with AN-BP (Age: *M* = 24.6, *SD* = 4.7; BMI: *M* = 16.4, *SD* = 1.4), 33 adults with BN (Age: *M* = 23.6, *SD* = 3.9; BMI: *M* = 22.0, *SD* = 3.9), and 30 healthy controls (Age: *M* = 23.9, *SD* = 3.5; BMI: *M* = 21.9, *SD* = 2.1).	EDE, SCID-5-CV.	Socially evaluated arithmetic task with somatic distractors.	SSAT.	Self-report (VAS), serum cortisol.	Age, sex, BMI, IQ.	Participants with BN had greater difficulty with proactive inhibition (i.e., slowing reaction time when the probability of a 'stop signal' was higher) under neutral conditions. This effect was not observed in AN-BP and control groups. Proactive inhibition was not significantly affected by stress in any group.<br><br>Reactive inhibition (i.e., reaction time latency to stop signal presentation) was not affected by acute stress across all participant groups.<br><br>BN and AN-BP groups consumed less kilocalories during an *ad libitum* food intake than the control group. *Ad*	Proactive inhibition was associated with heightened activity in the left SFG in the BN group relative to controls.<br><br>The BN group demonstrated increased activation of the right SFG under acute stress relative to the AN-BP group during proactive inhibition.<br><br>Acute stress was associated with diminished activation of the right vmPFC during reactive inhibition in the AN-BP group relative to controls.<br><br>Left SFG activation during proactive inhibition, and diminished vmPFC activation during reactive

			libitum consumption was not affected by stress exposure.	inhibition, predicted increased energy intake. These results were not affected by group or stress exposure.
Wonderlich et al. (2018) [5].	United States.	Refer to *Fischer et al. (2017).*	EMA-reported negative affect increased prior to binge-eating episodes and decreased following binge-eating. Positive affect decreased immediately prior to binge-eating episodes and increased following binge-eating.	Decreased activation of the bilateral vmPFC and right amygdala palatable food-cues under stress exhibited predicted significantly larger and faster increases in negative affect prior to binge-eating episodes.<br><br>Blunted activation of the ACC in response to highly palatable food-cues under stress predicted larger decreases in positive affect prior to binge-eating episodes.<br><br>Decreased activation of the left vmPFC and left amygdala under stress predicted larger and faster increases in positive affect following binge-eating episodes.

Note. ACC, Anterior cingulate cortex; AN-BP, anorexia nervosa binge-purge subtype; BN, bulimia nervosa; BMI, body mass index; CCUE, Cognitive Control Under Emotion; dlPFC, dorsolateral prefrontal cortex; dmPFC, dorsolateral prefrontal cortex; EDE, Eating Disorder Examination; EMA, ecological momentary assessment; M,

mean; MFG, middle frontal gyrus; NA: not applicable; SFG: superior frontal gyrus; SCID, structured clinical interview for DSM: SCID-I, structured clinical interview for DSM, clinical version; SCID-5-CV, Structured Clinical Interview for DSM-5, Clinical Version; SD, standard deviation; SSAT, Stop-Signal Anticipation Task; TSST, Trier Social Stress Test; VAS, visual analogue scale; vmPFC, ventromedial prefrontal cortex.

References

1. Collins B, Breithaupt L, McDowell JE, Miller LS, Thompson J, Fischer S. The Impact of Acute Stress on the Neural Processing of Food Cues in Bulimia Nervosa: Replication in Two Samples. *JOURNAL OF ABNORMAL PSYCHOLOGY*, 126(5), 540-551 (2017).
2. Dreyfuss MFW, Riegel ML, Pedersen GA *et al.* Patients with bulimia nervosa do not show typical neurodevelopment of cognitive control under emotional influences. *PSYCHIATRY RESEARCH-NEUROIMAGING*, 266, 59-65 (2017).
3. Fischer S, Breithaupt L, Wonderlich J *et al.* Impact of the neural correlates of stress and cue reactivity on stress related binge eating in the natural environment. *JOURNAL OF PSYCHIATRIC RESEARCH*, 92, 15-23 (2017).
4. Westwater ML, Mancini F, Gorka AX *et al.* Prefrontal Responses during Proactive and Reactive Inhibition Are Differentially Impacted by Stress in Anorexia and Bulimia Nervosa. *J Neurosci*, 41(20), 4487-4499 (2021).
5. Wonderlich JA, Breithaupt L, Thompson JC, Crosby RD, Engel SG, Fischer S. The impact of neural responses to food cues following stress on trajectories of negative and positive affect and binge eating in daily life. *JOURNAL OF PSYCHIATRIC RESEARCH*, 102, 14-22 (2018).
